# Nanoarchitectonics of tannic acid based injectable hydrogel regulate the microglial phenotype to enhance neuroplasticity for poststroke rehabilitation

**DOI:** 10.1186/s40824-023-00444-0

**Published:** 2023-10-31

**Authors:** Zongjian Liu, Shulei Zhang, Yuanyuan Ran, Huimin Geng, Fuhai Gao, Guiqin Tian, Zengguo Feng, Jianing Xi, Lin Ye, Wei Su

**Affiliations:** 1 Beijing Rehabilitation Hospital, Capital Medical University, Beijing, 100144 China; 2 School of Materials Science and Engineering, Beijing Institute of Technology, Beijing, 100081 China; 3 Department of Neurosurgery, Qilu Hospital of Shandong University, Shandong University, Jinan, Shandong 250012 China; 4 Beijing Tsinghua Chang Gung Hospital, School of Clinical Medicine, Tsinghua University, Beijing, 102218 China

**Keywords:** Tannic acid, Stroke, Injectable hydrogel, Microglia, Anti-inflammatory phenotype

## Abstract

**Background:**

Stroke is the second leading cause of mortality and disability worldwide. Poststroke rehabilitation is still unsatisfactory in clinics, which brings great pain and economic burdens to stroke patients. In this study, an injectable hydrogel in which tannic acid (TA) acts as not only a building block but also a therapeutic drug, was developed for poststroke rehabilitation.

**Methods:**

TA is used as a building block to form an injectable hydrogel (TA gel) with carboxymethyl chitosan (CMCS) by multivalent hydrogen bonds. The morphology, rheological properties, and TA release behavior of the hydrogel were characterized. The abilities of the TA gel to modulate microglial (BV2 cells) polarization and subsequently enhance the neuroplasticity of neuro cells (N2a cells) were assessed in vitro. The TA gel was injected into the cavity of stroke mice to evaluate motor function recovery, microglial polarization, and neuroplasticity in vivo. The molecular pathway through which TA modulates microglial polarization was also explored both in vitro and in vivo.

**Results:**

The TA gel exhibited sustainable release behavior of TA. The TA gel can suppress the expression of CD16 and IL-1β, and upregulate the expression of CD206 and TGF-β in oxygen and glucose-deprived (OGD) BV2 cells, indicating the regulation of OGD BV2 cells to an anti-inflammatory phenotype in vitro. This finding further shows that the decrease in synaptophysin and PSD95 in OGD N2a cells is effectively recovered by anti-inflammatory BV2 cells. Furthermore, the TA gel decreased CD16/iNOS expression and increased CD206 expression in the peri-infarct area of stroke mice, implying anti-inflammatory polarization of microglia in vivo. The colocalization of PSD95 and Vglut1 stains, as well as Golgi staining, showed the enhancement of neuroplasticity by the TA gel. Spontaneously, the TA gel successfully recovered the motor function of stroke mice. The western blot results in vitro and in vivo suggested that the TA gel regulated microglial polarization via the NF-κB pathway.

**Conclusion:**

The TA gel serves as an effective brain injectable implant to treat stroke and shows promising potential to promote poststroke rehabilitation in the clinic.

**Graphical Abstract:**

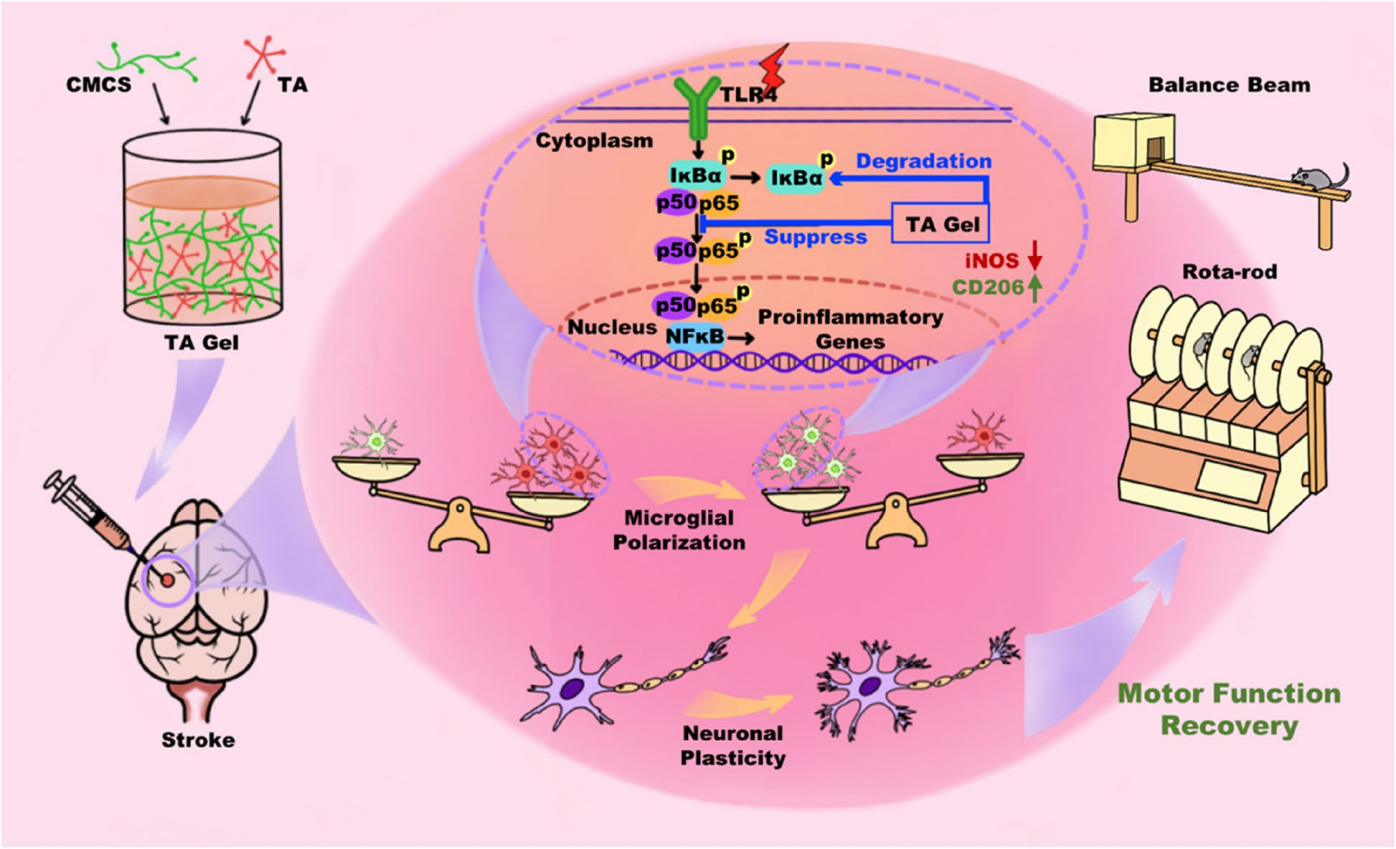

**Supplementary Information:**

The online version contains supplementary material available at 10.1186/s40824-023-00444-0.

## Introduction

Stroke is the second leading cause of mortality and disability worldwide, following heart diseas [1, 2]. However, poststroke rehabilitation is still unsatisfactory in clinics, which brings great pain and economic burdens to stroke patients [3]. Thus, developing a novel medical treatment for stroke rehabilitation, outside the confines of conventional therapies, is desirable. Recently, many strategies, such as angiogenesis, [4–6] neurogenesis, [7–9] and neuroplasticity [10], have been carried out to promote poststroke rehabilitation and have achieved significant progress. Microglia, the resident immune cells in the central nervous system, play a significant role in poststroke rehabilitation since they exert important effects on spontaneous recovery after stroke, including structural and functional reestablishment of neurovascular networks, neuroplasticity, neurogenesis, axonal remodeling, and blood vessel regeneration [11]. Our group has focused on the modulation of microglial polarization [12–15] in the subacute/chronic phase after stroke and first proposed a novel feasible strategy for poststroke rehabilitation that enhances neuroplasticity by regulating microglial polarization toward an anti-inflammatory phenotype [16].

As a natural polyphenol, tannic acid (TA) can be extracted from a variety of plants [17]. TA has attracted increasing attention because it is not only a versatile building block to construct multifunctional hydrogels with adhesive, stretchability, self-healing properties, and biodegradable ability [18, 19] but also has valuable pharmaceutical effects, including anti-inflammatory, antioxidative, and antibacterial effects [20]. Jafari, et. al. used TA to post treat the enzymatically crosslinked chitosan-alginate hydrogels and found that the hydrogel adhesiveness, antioxidant, and antibacterial properties, as well as the hydrogel cytocompatibility and cell proliferation, were significantly improved after TA treatment and were more suitable for biomedical applications [21]. Guan and Yao prepared a hydrogel with excellent injectable, self-healing, and adhesive properties by facile mixing of quaternary ammonium chitosan (QCS)/TA under physiological conditions [22]. Benefiting from the inherent antioxidative, antibacterial, and hemostatic abilities of TA and QCS, this hydrogel showed superior reactive oxygen species scavenging activity, broad-spectrum antibacterial ability, and rapid hemostatic capability that not only rapidly stopped the bleeding of arterial and deep incompressible wounds in mouse tail amputation, femoral artery hemorrhage, and liver incision models but also significantly accelerated wound healing in a full-thickness skin wound model. These results indicate the comprehensive utilization of the pharmaceutical effects of TA, and its advantages in hydrogel preparation enable the fabrication of promising biomedical materials with satisfactory properties.

Considering the outstanding anti-inflammatory properties of TA, as mentioned above [21, 22], we proposed that TA gel might be able to modulate microglia to express an anti-inflammatory phenotype after stroke and has the potential to treat stroke and promote poststroke rehabilitation. However, there are some restrictions in drug delivery for stroke [23]. With the gradual recovery of the blood–brain barrier (BBB) in the subacute/chronic phase, drugs with intravenous injection can hardly pass the BBB and have very low bioavailability in the brain [24]. Although intracranial injection can directly deliver the drug into the brain, frequent cerebrospinal fluid circulation can quickly remove the delivered drug from the brain [25, 26]. Hence, the treatment of stroke in the subacute/chronic phase requires a drug carrier that can not only directly deliver the drugs to the brain, but also maintain sustainable release on-site. On the other hand, stroke leads to tissue damage and the formation of an irregular cavity, [4, 27] which represents a potential location for drug delivery [28–30]. Since the cavity is irregular, an injectable hydrogel that is delivered in liquid form, freely fills the irregular cavity, forms a hydrogel in situ, and sustainably releases the drug on-site via diffusion, is the most suitable delivery system for stroke in the subacute/chronic phase [23].

In this study, TA solution was mixed with carboxymethyl chitosan (CMCS) to obtain an injectable hydrogel (TA gel) where TA acts as not only the building block but also the bioactive drug. We found that the TA gel effectively regulated microglial polarization toward an anti-inflammatory phenotype when it was applied to microglia soon after oxygen–glucose deprivation (OGD), and anti-inflammatory microglia enhanced the synaptic plasticity of neurons in vitro. Subsequently, the TA gel was implanted into the cavity of stroke mice induced by the photothrombotic (PT) method. The PT model can cause a cavity in the cortex, which reduces the difficulty of intracranial injection. Immunofluorescence (IF) staining suggested that the TA gel can also regulate microglial polarization toward an anti-inflammatory phenotype and further enhance neuroplasticity in vivo. The ethological tests, including beam balance and rotarod, showed the significant recovery of motor function of stroke mice by TA gel. Additionally, molecular biological analysis indicated that the TA gel plays an anti-inflammatory role and regulates microglial polarization via the classical NF-κB signaling pathway. Consequently, TA gel, due to its easy fabrication and significant therapeutic effect, shows promising potential in the treatment of subacute stroke to promote poststroke rehabilitation.

## Materials and methods

### Materials

CMCS (MW 20 − 30 kDa, degree of carboxylation ∼80%, Beijing Solarbio Science & Technology Co., Ltd., CHN), TA (J&K Scientific Ltd., CHN), Cy5 (RuixiBio, CHN), phosphate buffered saline (PBS, Solarbio, CHN), normal saline (0.9%, China Resources Double-crane Pharmaceutical Co., Ltd., CHN), paraformaldehyde (PFA, 4%, Solarbio, CHN), sodium pentobarbital (Merck, Germany), and rose bengal (Sigma-Aldrich, Germany) were used.

Primary antibodies for IF staining:

Rabbit anti-IBA-1 (ab178846, Abcam, UK), mouse anti-iNOS (MA5-17,139, Invitrogen), goat anti-CD206 (AF2535, R&D), mouse anti-PSD95 (ab2723, Abcam, UK), and rabbit anti-Vglut1 (GTX133148, GeneTex, USA) were used.

Primary antibodies for WB:

Rabbit anti-CD206 (24,595, CST, USA), rabbit anti-iNOS (GB113965, Solarbio, CHN), mouse anti-p65 (GB12142, Solarbio, CHN), rabbit anti-p-p65 (GB113882, Solarbio, CHN), rabbit anti-IĸBα (GB111509, Solarbio, CHN), rabbit anti-p-IĸBα (AF2002, AFFinity, USA), rabbit anti-Synaptophysin (GB11814, Solarbio, CHN), rabbit anti-PSD95 (GB11277, Solarbio, CHN), rabbit anti-CD16 (80,006, CST, USA), rabbit anti-IL-1β (GB11113, Solarbio, CHN), rabbit anti-TGF-β (GB111876, Solarbio, CHN), rabbit anti-TLR4 (GB11519, Solarbio, CHN), rabbit anti-LaminB1 (GB111802, Solarbio, CHN), and mouse anti-GAPDH (GB15002, Solarbio, CHN) were used.

### Preparation of the TA gel

TA was dissolved in deionized water to obtain a TA solution at a concentration of 6 mg/mL. Then, 50 mg of CMCS was added to 500 μL of TA solution to obtain a TA gel. The gel was centrifuged at 4000 rpm for 5 min to remove air bubbles.

### In vitro characterization of the TA gel

#### Gelation

The gelation time of the TA gel was determined by the inverted vial method. The TA solution was added to a small glass vial containing CMCS powder and mixed well to start the timer. Thirty seconds later, the vial was inverted, and the solution no longer flowed to suggest that a gel had formed. The time taken for this process is the gelation time.

#### Morphological observation

The cross-sectional morphology of the TA gels was observed by scanning electron microscopy (SEM, Regulus S8230). The gel was freeze-dried, immersed in liquid nitrogen and quickly cut for SEM observation. The samples were gold sprayed to improve the electrical conductivity of the samples prior to observation.

#### IR measurement

The as-prepared hydrogels were freeze-dried and characterized by FT-IR (Shimadzu IRTrace-100).

#### Degradation

The TA gel was freeze-dried and weighed, and the mass was recorded as m_0_. The TA gel was placed in a 10 mL centrifuge tube containing 8 mL of PBS solution. The centrifuge tubes were placed in a water bath shaker and gently shaken at 37℃. The sample was removed at the time interval required for the experiment, and freeze-dried, and the sample mass was weighed and recorded as m_x_. The degradation rate (DR) of the TA gel was calculated by the following equation.

$$\mathrm{DR }= ({\mathrm{m}}_{0}- {\mathrm{m}}_{\mathrm{x}})/{\mathrm{m}}_{0} \times 100\mathrm{\%}$$

#### In vitro release of TA

The in vitro release of TA was measured by a UV–Vis spectrophotometer (UV-1800). First, the absorbance of a series of TA solutions in a concentration gradient was measured to establish a standard curve (Fig. S7). Then, 1 mL of TA gel was completely submerged in a 50 mL centrifuge tube containing 10 mL of PBS solution and gently shaken in a bath shaker at 37℃. Then, 0.5 mL of release solution was removed at predetermined time points and supplemented with 0.5 mL of fresh PBS solution. The solution was further measured by a UV–Vis spectrophotometer (UV-1800) to obtain the in vitro release plot of TA.

### In vitro cell experiments

#### Biocompatibility of the TA gel

N2a cells were cultured with the medium extracted from the TA gel to evaluate the biocompatibility of the TA gel. TA gel (0.1 mL) was prepared in a glass vial, and then 5 mL of culture medium was added to the vial. The TA gel was extracted at 37℃ for 24 h to obtain the TA gel extracted solution (TAG). N2a cells were cultured in 96-well plates (1 × 10^4^ cells/well) for 24 h. The medium was then replaced with different TAGs (0%, 50%, 100%) diluted with normal culture medium. Cells were cultured in a humidified environment at 37℃ and 5% CO_2_ for 24 h and 48 h, respectively. Then, cell viability was measured by live and dead cell assays according to the manufacturer’s instructions (CA1630, Solarbio, CHN) as well as CCK-8 assays. Fluorescence images were acquired by fluorescence imaging microscopy (Olympus BX51, Japan).

#### The modulation of microglial phenotype by TA gel in vitro

BV2 (mouse microglial) was purchased from Wuhan Pronoxyl Life Sciences Co. Cells were cultured in DMEM (HyClone) supplemented with 10% fetal bovine serum (Gibco, USA) and 1% penicillin/streptomycin (1 million U/L each) in a 37℃, 5% CO_2_ incubator (SANYO (XD-101), Japan). Cells were cultured until they reached 70% or more confluence under the microscope, with the culture medium changed every two days and passaged every three days.

BV2 cells were seeded in 96-well plates (1 × 104 cells/well) and cultured with different TAGs (0%, 25%, 50%, 100%) for 24 h. Then, cell viability was measured by CCK-8 assay, and cytotoxicity was measured by LDH assay.

BV2 cells were first cultured with sugar-free medium and then placed in an OGD (glucose and oxygen deprivation) tank filled with gas (95% N2/5% CO_2_) for 10 min. The tank with BV2 cells was closed and placed in the incubator for 3 h without sugar and oxygen. Then, the OGD tank was removed, and the sugar-free medium was changed to sugar-containing medium. As soon as oxygen and glucose were replenished, TAG (50%) was added. BV2 cells were further cultured for 24 h for examination.

The expression of iNOS and CD206 in BV2 cells was first characterized by immunofluorescence staining (Olympus BX51, Japan). Furthermore, cells were collected and made into whole cell lysates. The expression levels of CD16, CD206, IL-1β, and TGF-β in BV2 cells were measured by western-blot (WB) analysis. Finally, the expression levels of TLR4, phosphor-p65, p65, phosphor-IκBα, and IκBα in BV2 cells were also measured by WB. Then, the cytoplasm and nucleus were separated, and the expression of p65 in the cytoplasm and nucleus was measured by WB.

#### Synaptic plasticity modulated by BV2 cells in vitro

Four groups of BV2 cells, BV2, BV2 + 50% TAG, OGD BV2, and OGD BV2 + 50% TAG, were cultured in the upper chamber of the Transwell system for 12 h. The OGD operation lasted for 3 h. Then, the culture media were removed, and BV2 cells were washed with fresh medium three times to completely remove 50% TAG.

Two groups of N2a cells, N2a and OGD N2a, were seeded on the lower plate of the Transwell system and cultured in the mixing medium (BV2:N2a = 1:1). The OGD operation lasted for 3 h. Four BV2 groups and two N2a groups were assembled in pairs to form eight complete Transwell systems. BV2 and N2a cells were cocultured in Transwell systems for 24 h to investigate the influences of BV2 cells on N2a cells. Viability was assessed by CCK-8 assay, and the expression of synaptophysin and PSD95 proteins was measured by WB.

### In vivo experiments

#### Animals

All in vivo studies were approved by the Animal Experiment Ethics Committee of Beijing Rehabilitation Hospital, Capital Medical University. All animal care, housing, surgical, and anesthetic procedures were performed in accordance with the Regulation for the Administration of Affairs Concerning Experimental Animals of China. Animals were housed five- per- cage at a standard temperature (22 ± 1℃) with ad libitum access to food and water. Male C57BL/6 J mice (aged 12 weeks, 26.0 ± 1.5 g) were used in the experiments.

#### Photothrombotic stroke model

The mice were anesthetized with isoflurane (4%, 3 min), the head was shaved, and the scalp was sterilized. Then, the mice were fixed on a Mouse Stereotaxic, and a midline incision was made along the rostrocaudal axis to separate the skull from the skin and expose the skull. The mice were injected with the rose red solution at a concentration of 10 mg/ml at a dose of 10 μL/g through the tail vein and circulated for 7 min. A laser with a wavelength of 561 nm and a power of 40 mW was used to irradiate the upper left side of the skull of mice for 15 min with a view to forming a thrombus. The skin on the head of the mice was sutured and placed on a heating pad (37℃) for 5–10 min.

After the mice recovered from anesthesia, they were placed in a new cage and supplemented with food and water as needed. In this case, the sham-operated group was injected with the same dose of PBS solution through the tail vein, and the rest of the steps were the same as above.

#### Injection of TA gel

On the 7th day after stroke, all mice were divided into 5 groups based on ethological data. The five groups were sham-operated (Sham), untreated (Stroke), TA gel (TAG), TA solution (TAS) and CMCS solution. Mice were anesthetized with isoflurane (4%, 3 min), and 3 µL of TA gel, TA solution and CMCS solution were injected into the cavity of the stroke model using a syringe, whereas the sham and stroke groups were left untreated.

#### In vivo imaging of TA gels

On the 7th day after stroke, 3 µL of Cy5-labeled TA gel was injected into the stroke cavity of the mice and photographed on the 7th, 9th, 14th, 17th and 21st days after stroke by a small animal fluorescence imaging system (IVIS® Lumina III, PerkinElmer, USA) to observe the degradation of the TA gel in the mice.

#### Ethology assessment

Rotarod Test: Mice were trained on a rotarod (Panlab Rotarod LE8505) at 10 rpm for 3 days prior to the former test. During the test, the speed of the rotarod was accelerated uniformly from 4 to 40 rpm over 5 min, and the time when the mouse fell off the rotating axis was recorded.

Balance Beam Test: Motor coordination was quantified by counting the number of times that the right hind paw slid off the beam (approximately 80 cm long, 1.5 mm wide and 100 cm off the ground) during the experiment. Acquired training was performed for 3 days prior to the formal test.

The test was repeated three times for each mouse.

#### In vivo characterization of microglial polarization and neuroplasticity

On Day 21 after stroke, 1% sodium pentobarbital was injected at a dose of 0.3 mL to anesthetize the mice. As soon as the heart was exposed, it was perfused with 0.9% saline from the left ventricle and then 4% PFA. The brain tissue was isolated and preserved in PFA (4%) for fixation. Microglial polarization in vivo was characterized by WB and IF staining, whereas neuroplasticity in vivo was measured by WB, IF staining and Golgi staining (Servicebio, GP1152).

Brain tissue from the marginal zone of cortical brain infarction was collected, and whole cell lysates were prepared. WB was performed to detect the expression of iNOS and CD206 to investigate microglial polarization in vivo, synaptophysin and PSD95 to explore synaptic plasticity in vivo, and phos-p65, p65, phos-IĸBα, and IĸBα to demonstrate the role of NF-κB pathway in microglial polarization.

Three-millimeter- thick slices of brain tissue were embedded in paraffin, and 4 µm slices were cut out for subsequent experiments using a microtome (Leica RM2235, Germany). IF staining was performed by adding a primary antibody to the tissue sections and incubating overnight at 4℃. A secondary antibody working solution corresponding to the source of the primary antibody was then added and incubated for 30 min at 37℃, protected from light. Nuclei were then stained with DAPI for 10 min (protected from light). Finally, fluorescence scanning of the slices was performed using digital slice scanners (KF-PRO-020, KFBIO, Ningbo Jiangfeng Bioinformatics Co., Ltd.).

### Statistics

For the immunofluorescence staining results, five fields of view (magnification 400) of the target area were selected for each image, and the positive area was counted using ImageJ software. Statistical analysis was performed using GraphPad Prism software (version 5.01). The results are expressed as the mean ± standard deviation (SD). Student’s t test was used to test the difference between two groups. One-way ANOVA (one-way analysis of variance) was used to test for differences between multiple groups. In all analyses, *p* < 0.05 was considered statistically significant, where *p < 0.05, **p < 0.01 and ***p < 0.001.

## Results

**Fig. 1:**
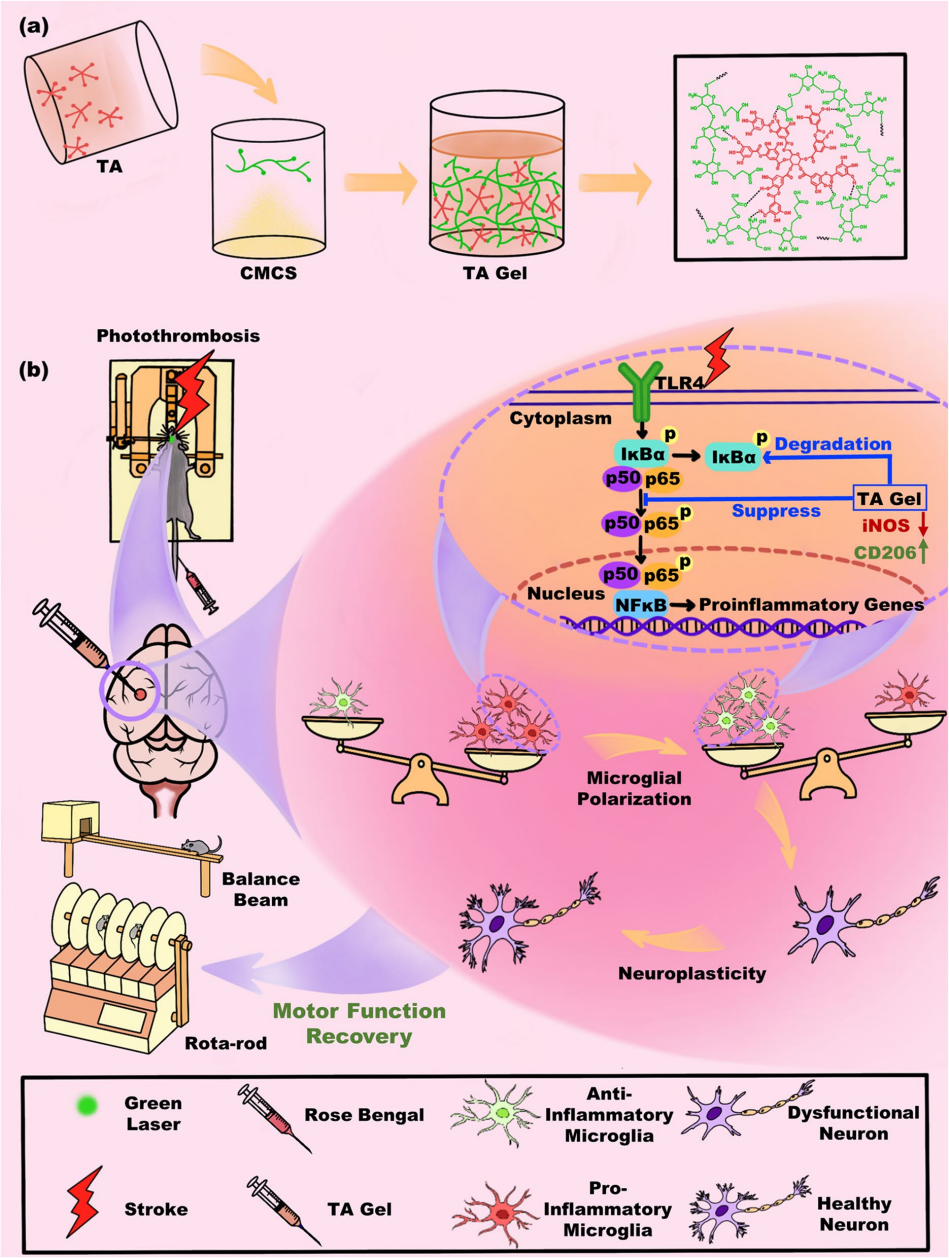
**a** The preparation protocol and **b** the proposed mechanism by which TA gel promotes poststroke rehabilitation

Figure 1 shows the proposed mechanism by which TA gel promotes poststroke rehabilitation. The chemical reaction equation of the TA gel was depicted in Fig. S1. The hydrogel was formed by simply mixing CMCS and TA. Since TA usually acts as an H-donor and CMCS could be an H-receptor, the reaction can be carried out very fast under physiological pH 7.4 at body temperature without any external stimulus. TA is not only a necessary building block for hydrogel preparation but also an effective drug that is used to enhance neuroplasticity for poststroke rehabilitation. The TA gel can sustainably release TA to the peri-infarct area in vivo. It is proposed that TA, as an anti-inflammatory drug, can modulate microglial polarization towards an anti-inflammatory phenotype via the classical NF-κB pathway. Subsequently, anti-inflammatory microglia can play a vital role in enhancing neuroplasticity to promote functional recovery after stroke.

**Fig. 2:**
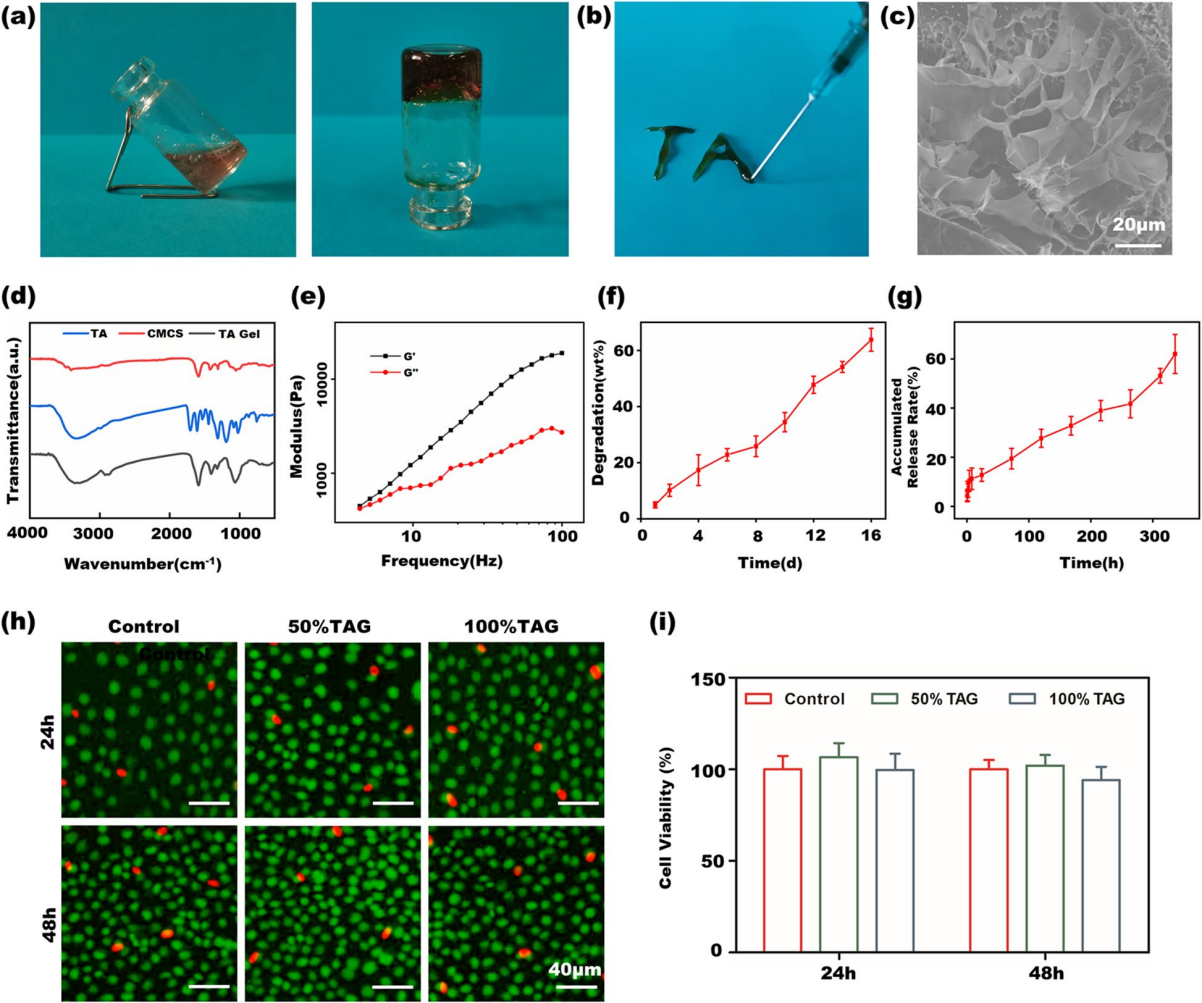
In vitro characterization of the TA gel **a** hydrogel formation demonstrated by inverted vital method; **b** the TA gel injected out from a syringe; **c** the morphological observation by SEM; **d** IR spectra of TA, CMCS and TA gel; **e** rheological measurement of the TA gel; **f** in vitro degradation of the TA gel; **g** in vitro release behavior of the TA; **h** N2a cell viability characterized by live/dead assay; **i** N2a cell viability measured by CCK-8

The in vitro characterization of the TA gel is shown in Fig. 2. Figure 2a shows the successful formation of the TA hydrogel by the inverted vial method. Figure 2b shows that the TA gel could be injected from a syringe, indicating that it could be intracranially delivered into the brain by the syringe. The porous structure of the TA hydrogel is depicted by the SEM image in Fig. 2c, and gold spraying was used to improve the electrical conductivity of hydrogel sample [31, 32]. Figure 2d shows the FT-IR spectra of TA, CMCS and the TA gel. TA and CMCS showed strong, broad bands of − OH stretching vibration at 3480 and 3430 cm^−1^, respectively. In the spectrum of the TA gel, the − OH absorption peak shifted to 3410 cm^−1^, which demonstrated the presence of hydrogen bonds between them. The IR result indicated that TA gel is formed by the hydrogel bond interactions between TA and CMCS. The rheological properties of the TA gel are shown in Fig. 2e. The low modulus ranging from 10^3^ to 10^4^ Pa makes the gel injectable to a certain extent [33]. The degradation and TA release behavior in vitro are shown in Fig. 2f and g. The weight loss plot of the TA gel during degradation showed that the TA gel gradually lost approximately 60% of its weight for 16 days, whereas the in vitro release behavior showed that TA can be sustainably released from the hydrogel for approximately two weeks. The biocompatibility and cytotoxicity of the TA gel is shown in Figs. 2h and S2 by live/dead cell assay. TA gel (TAG) was extracted by the culture medium, and the extracted culture medium was diluted with different volumes of culture medium and used to culture N2a cells. On the one hand, the number of live cells in the different dilutions was similar to that cultured in complete culture medium, indicating the satisfactory biocompatibility of the TA gel. On the other hand, both different dilutions and complete cell medium possessed very few dead cells, implying the low cytotoxicity of the TA gel. The biocompatibility of the TA gel is further characterized by the CCK-8 assay in Fig. 2i, which is consistent with the results of the live/dead cell assay in Fig. 2h.

**Fig. 3:**
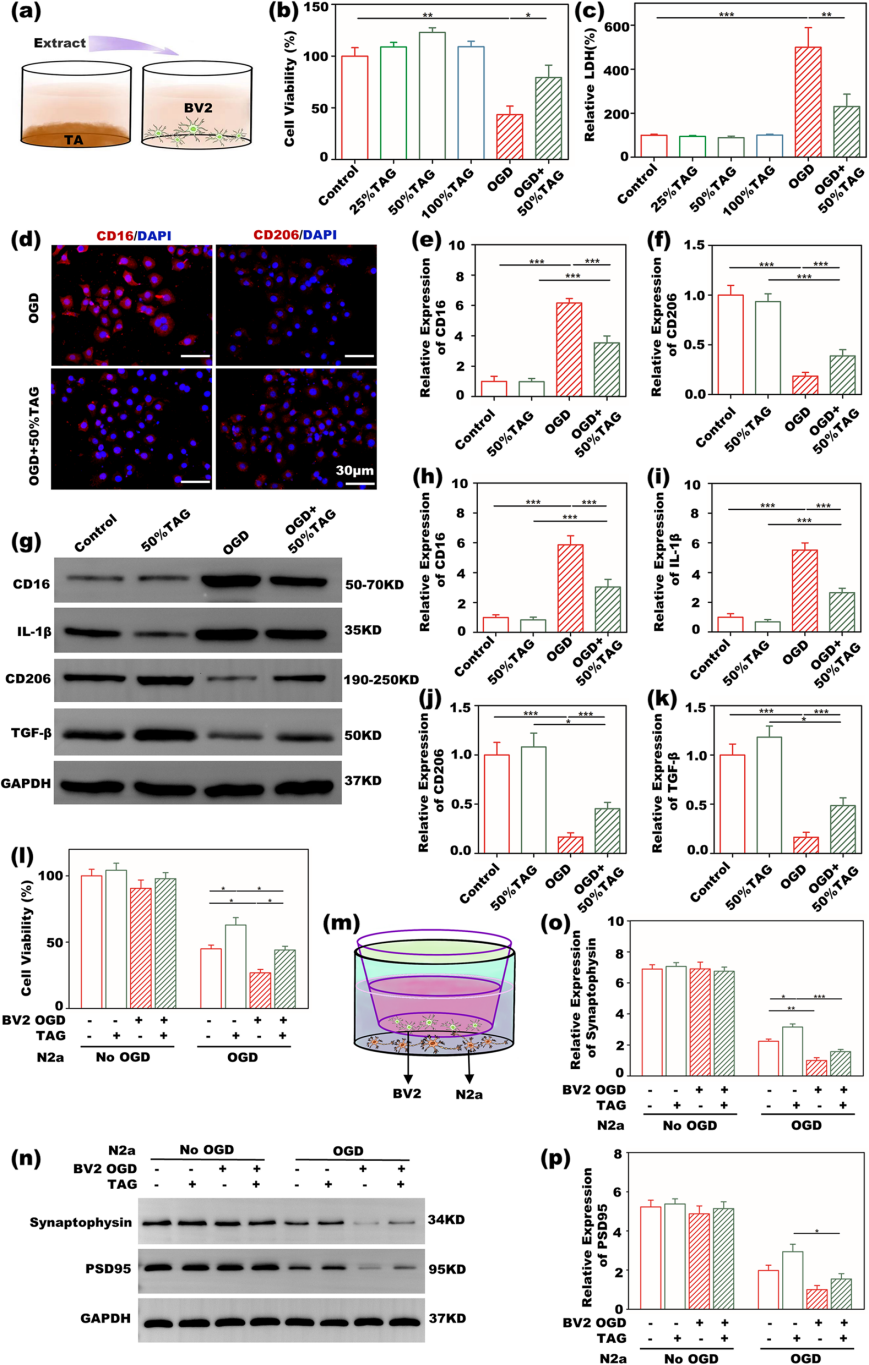
In vitro cell culture **a** schematic diagram of BV2 cell culture; **b** BV2 cell viabilities treated with different TAG dilutions before and after OGD; **c** cytotoxicity by LDH measurement before and after OGD; **d** IF images of CD16 and CD206 staining in BV2 cells after OGD; semiquantitative results of **e** CD16 and **f** CD206 staining; **g** representative WB strips of CD16, IL-1β, CD206 and TGF-β expression in microglia before and after OGD; quantitative WB results of **h** CD16, **i** IL-1β, **j** CD206 and **k** TGF-β; **l** N2a cell viabilities cocultured with BV2 cells before and after OGD; **m** schematic diagram of N2a and BV2 cell coculture; **n** representative WB stripes of synaptophysin and PSD95 expression in N2a coculture with BV2 cells before and after OGD; quantitative WB results of **o** synaptophysin and **p** PSD95

Figure 3a shows schematics of BV2 cell culture. Figure 3b shows that cells treated with different concentrations of the extracted medium had a proliferation rate similar to that of cells treated with complete culture medium, indicating good biocompatibility of the TA gel with BV2 cells. After OGD, the viability of BV2 cells in the control treated with complete culture medium markedly decreased, whereas the 50% TAG group significantly recovered the viability of OGD BV2 cells. Figure 3c shows that the TAG groups also possessed low LDH release similar to that of the control, implying the low cytotoxicity of the TA gel. Correspondingly, LDH release in the control group greatly increased after OGD, while treatment with TAG significantly suppressed LDH release. The IF images in Figs. 3d and S3 show that BV2 cells, as the control, expressed a high level of the proinflammatory protein marker CD16 and a low level of the anti-inflammatory protein marker CD206 after OGD. However, TAG significantly decreased the expression of CD16 and increased the expression of CD206 in OGD BV2 cells. The semiquantitative results of IF images are shown in Fig. 3e and f, which clearly showed lower CD16 and higher CD206 expression in the TAG group than in the control after OGD, with significant differences. The WB strips and quantitative results are shown in Fig. 3g-k, which showed similar results for IF staining. The TAG group also exhibited lower CD16 and higher CD206 expression after OGD than those in the control group, with significant differences. Thus, the above results clearly demonstrated our proposal that the TA gel can modulate BV2 cell polarization toward an anti-inflammatory phenotype. Furthermore, as IL-1β and TGF-β are typical proinflammatory and anti-inflammatory factors secreted by BV2 cells, respectively, lower IL-1β expression and higher TGF-β expression in the TAG group further implied the ability of the TA gel to regulate the anti-inflammatory polarization of microglia. The N2a cells were cocultured with BV2 cells in the Transwell system as shown in Fig. 3l. Normal BV2 cells and OGD BV2 cells were first cultured with normal culture medium and TAG, respectively. Then, four groups BV2, BV2 + TAG, OGD BV2, and OGD BV2 + TAG, were cocultured with normal N2a and OGD N2a. The WB strips and quantitative results are available in Fig. 3m-o. The four groups cocultured with normal N2a cells did not show any significant differences in the expression of synaptophysin and PSD95. However, significant differences were found for OGD N2a cells. The lowest expression of the two proteins was observed when OGD N2a cells were cocultured with OGD BV2 cells. This result indicated that stroke significantly leads to the apoptosis of neurons in vitro*,* as synaptophysin and PSD95 are two major proteins involved in synaptic plasticity. When OGD N2a cells were cocultured with OGD BV2 + TGA cells, the expression of synaptophysin and PSD95 significantly increased compared with that in OGD BV2 cells. Additionally, BV2 + TAG also caused OGD N2a cells to express more synaptophysin and PSD95 than BV cells. These results implied that TA gel can exert a beneficial influence on enhancing neuroplasticity in vitro.

**Fig. 4:**
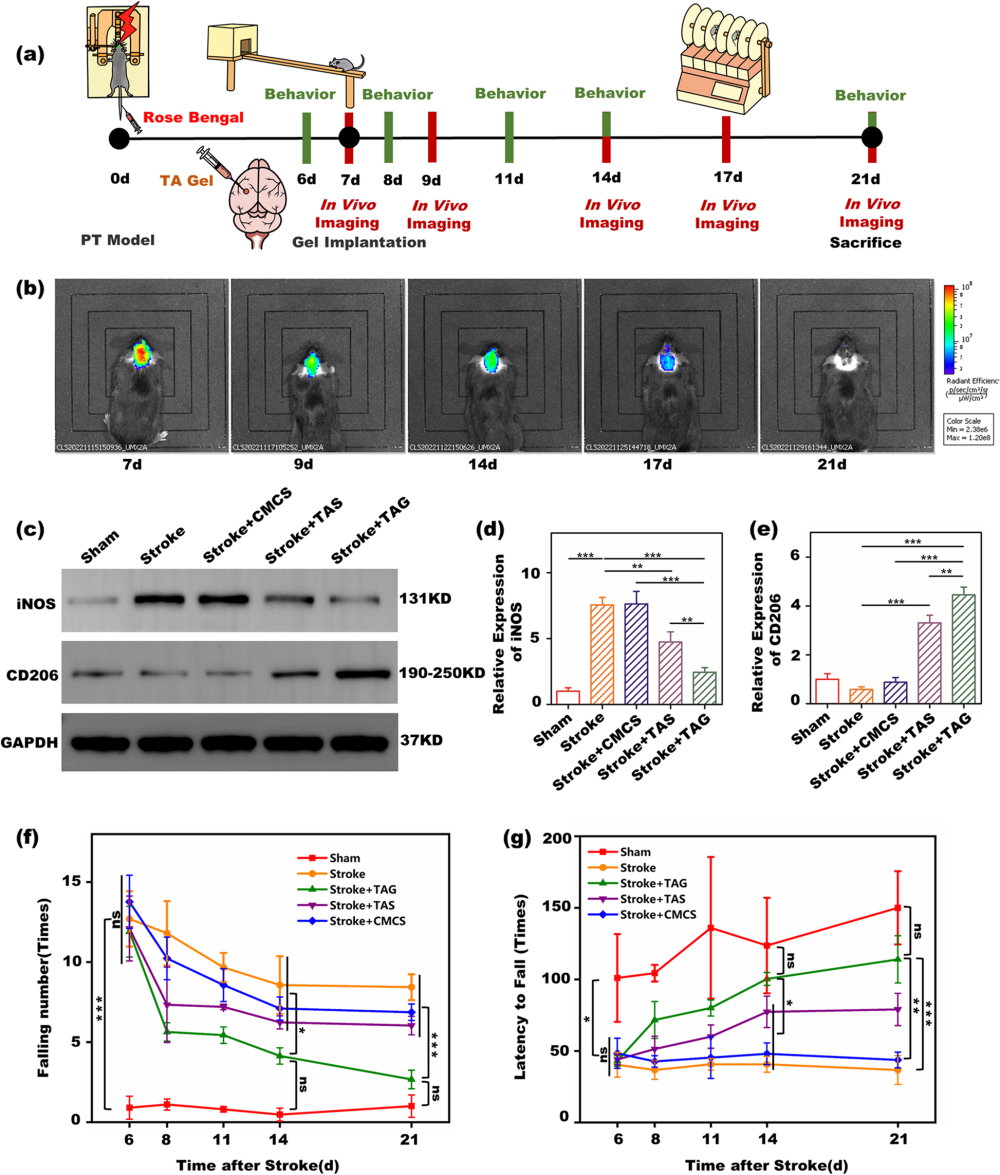
In vivo experiment of TA gel in the mouse **a** the protocol of in vivo experiment; **b** in vivo fluorescence images of the TA gel at different implantation time points; **c** representative WB stripes of iNOS and CD206 expressions in the stroke mice; quantitative WB results of **d** iNOS and **e** CD206; ethological results of **f** balance beam and **g** rotarod tests

Figure 4a depicts the protocol of the in vivo test in this study. The TA gel was implanted into the infarct cavity of the stroke mouse on the 7th day after the stroke induced by the PT model. The ethological tests were carried out on at the 6th, 8th, 11th, 14th, and 21st days to observe the recovery of motor function of the stroke mouse by TA gel. The mice were sacrificed on the 21st day for WB and IF measurements. Figure 4b shows the in vivo images of the implanted gel. As shown in Fig. S4, the fluorescent molecule, Cy5 was covalently bonded to the TA gel; thus, it could be visually detected by a small animal fluorescence imaging system. The immunofluorescence spectra and the SEM image of the fluorescence TA gel are shown in Figs. S5 and S6, respectively. As soon as the fluorescent TA gel was implanted in the cavity, a strong fluorescence signal could be observed, indicating the successful intracranial delivery of the gel. The intensity of the signal gradually weakened with the implantation time, implying the gradual degradation of the gel in vivo. The signal was still present on the 18th day, whereas it could hardly be detected on the 21st day. This implied that the TA gel can stay at least 12 days in vivo to sustainably release tannic acid to enhance neuroplasticity. Figure 4c-e tentatively shows the ability of the TA gel to regulate the polarization of microglia in vivo. The sham group had a relatively low level of both iNOS and CD206 expression, as the microglia did not polarize in the healthy mice, whereas stroke mice exhibited the highest iNOS and the lowest CD206 expression, indicating the polarization of the microglia toward a proinflammatory phenotype. Nevertheless, both the TA solution (TAS) and TA gel (TAG) groups exhibited significantly lower iNOS and higher CD206 expression than the stroke group, implying that tannic acid has the ability to regulate the polarization of microglia toward an anti-inflammatory phenotype. Between the two TA groups, TAG had even better performance with regard to regulating the polarization of microglia than TAS, hinting that the TA gel has promising potential to enhance neuroplasticity and recover the motor function of stroke mice. However, the CMCS solution group had similar iNOS and CD206 expression as the stroke group and did not show any ability to modulate microglia to express an anti-inflammatory phenotype. Figure 4f and g show the results of the balance beam and rotarod measurements to evaluate the recovery of motor function in stroke mice by TA gel. In the beam balance test, stroke markedly increased the falling number with treatment time. Although it has a similar baseline with other groups including the stroke, the TAS and the CMCS groups before receiving gel administration at the 6th day, the TAG group began to show significant differences with other groups starting on the 11th day and approached that of the sham group on the 21st day. The TAS group also had certain positive influences on decreasing the falling number, whereas the CMCS group did not show any positive influences. In the rotarod test, the opposite tendency was observed. Stroke greatly decreased the latency to fall of mice, while treatment with TA gel gradually increased it with treatment time. The TAG group also began to show significant differences from the other groups beginning on the 11th day. On the 21st day, the latency to fall of the TAG closely approached that of the sham group, and no significant difference was found between them. The TAS group also has positive influences to some extent, whereas the CMCS group does not show any positive influences. Consequently, these ethological results indicated the satisfactory recovery of motor function of stroke mice by TA gel.

**Fig. 5:**
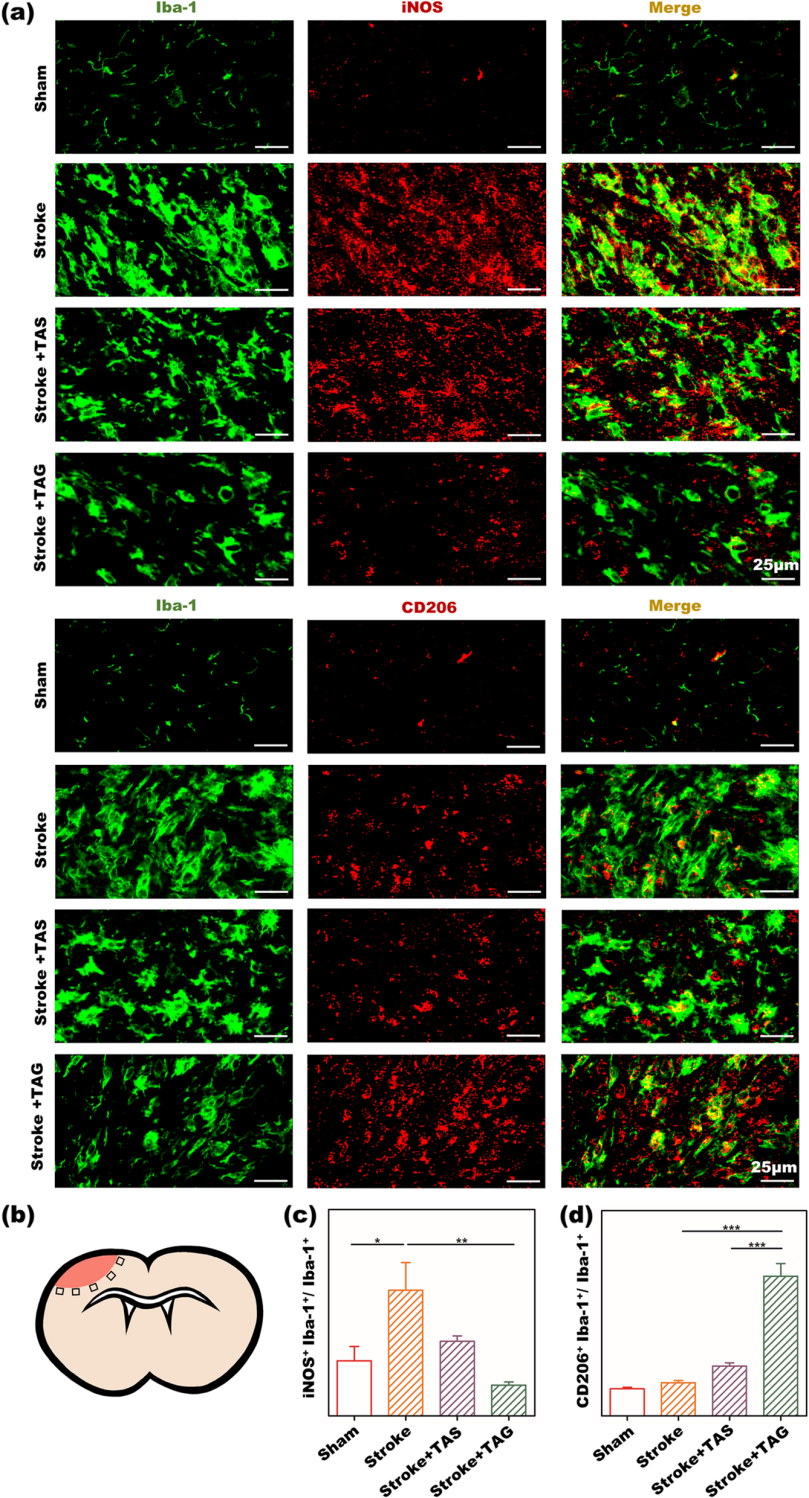
In vivo microglial polarization modulated by the TA gel. **a** IF images of IBA-1/iNOS and IBA-1/CD206 staining in the peri-infarct zone of stroke mice; **b** the representative five areas for quantitative calculation of IF stains; the quantitative results of **c** IBA-1^+^iNOS^+^/IBA-1^+^ and **d** IBA-1^+^CD206^+^/IBA-1^+^ percentage

Figure 5 shows images of IBA-1/iNOS and IBA-1/CD206 staining of the peri-infarct area in stroke mice. IBA-1 is a specific marker of activated microglia, thus, the colocalization of IBA-1^+^iNOS^+^ represents proinflammatory microglia, whereas that of IBA-1^+^CD206^+^ corresponds to anti-inflammatory microglia. Figure 5a shows that IBA-1 expression was very weak in the sham group, as most of the microglia remained at rest in the healthy mice. IBA-1 expression becomes much stronger after stroke, indicating the activation of microglia in the peri-infarct area. The TAG group expresses a slightly lower IBA-1 expression than that of the TAS and the control groups, showing that the TAG group can suppress the activation of the microglia to some extent. Furthermore, the semiquantitative results of the colocalization of IBA-1^+^iNOS^+^ and IBA-1^+^CD206^+^ are depicted in Fig. 5c and d. The stroke group expressed the highest colocalization of IBA-1^+^iNOS^+^ and the lowest colocalization of IBA-1^+^CD206^+^, implying that the microglia in this group mainly expressed the proinflammatory phenotype. In contrast, the TAG group exhibited the lowest colocalization of IBA-1^+^iNOS^+^ and the highest colocalization of IBA-1^+^CD206^+^, indicating that the microglia in the peri-infarct area preferentially polarized toward an anti-inflammatory phenotype in this group. The colocalization of both IBA-1^+^iNOS^+^ and IBA-1^+^CD206^+^ cells in the TAS group was moderate, showing that TAS had little effect on modulating microglial polarization.

**Fig. 6:**
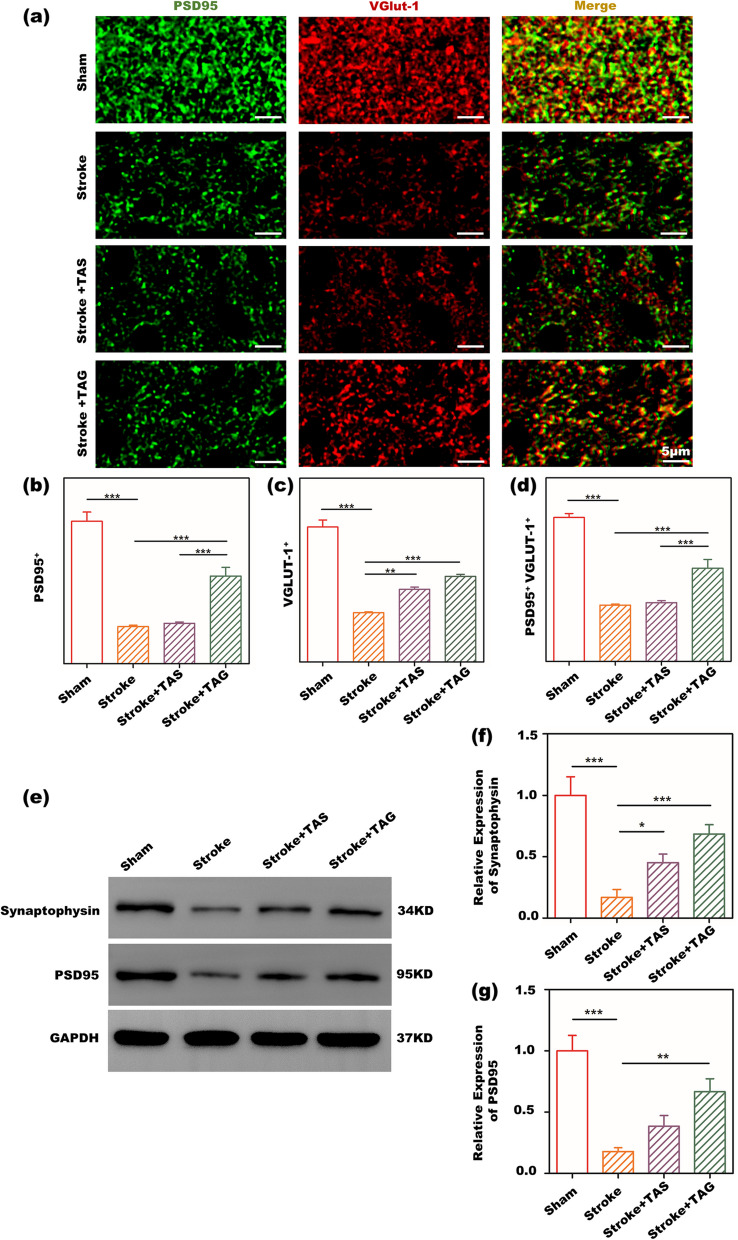
In vivo synaptic plasticity modulated by TA gel **a** IF images of PSD95 and Vglut1 staining in the peri-infarct zone of stroke mice; the quantitative results of **b** PSD95^+^, **c** Vglut1^+^ and **d** PSD95^+^Vglut1.^+^ in IF images of PSD95 and Vglut1 staining; **e** representative WB bands of synaptophysin and PSD95 expression in the peri-infarct area in vivo; quantitative WB results of **f** synaptophysin and **g** PSD95

Figure 6a-d shows the synaptic plasticity of stroke mice evaluated by double staining for PSD95 and Vglut1. Since PSD95 and Vglut1 are typical postsynaptic and presynaptic markers, respectively, the colocalization of PSD95^+^Vglut1^+^ can indicate the synaptic connection. Spontaneously, better synaptic connection means enhanced synaptic plasticity, which may further lead to better functional recovery. A similar tendency was found for PSD95 and Vglut1. The sham group had the highest PSD95 and Vglut1 expression, indicating perfect synaptic networks in healthy mice. The TAG group possesses the second place, implying the enhancement of the synaptic plasticity by the TA gel after stroke. TAS is third, showing that the TA solution has little positive influence on enhancing synaptic plasticity. Not surprisingly, the stroke group showed the lowest PSD95 and Vglut1 expression due to neuronal apoptosis caused by ischemic stroke. Furthermore, the colocalization of PSD95^+^Vglut1^+^ showed the same trend as PSD95 and Vglut1 expression: the sham group had the best synaptic connections, followed by the TAG group. The TAS and stroke rank third and last, respectively. Figure 6e-g show the WB results of synaptophysin and PSD95 expression in the peri-infarct zone. Synaptophysin and PSD95 are two representative proteins related to synaptic plasticity. The sham group had the highest expression of these two proteins, followed by the TAG group. The TAS and stroke possess the last two places in turn. Hence, the colocalization of PSD95^+^Vglut1^+^ as well as the WB results of synaptophysin and PSD95 expression demonstrated that TA gel treatment can effectively enhance synaptic plasticity in vivo.

**Fig. 7:**
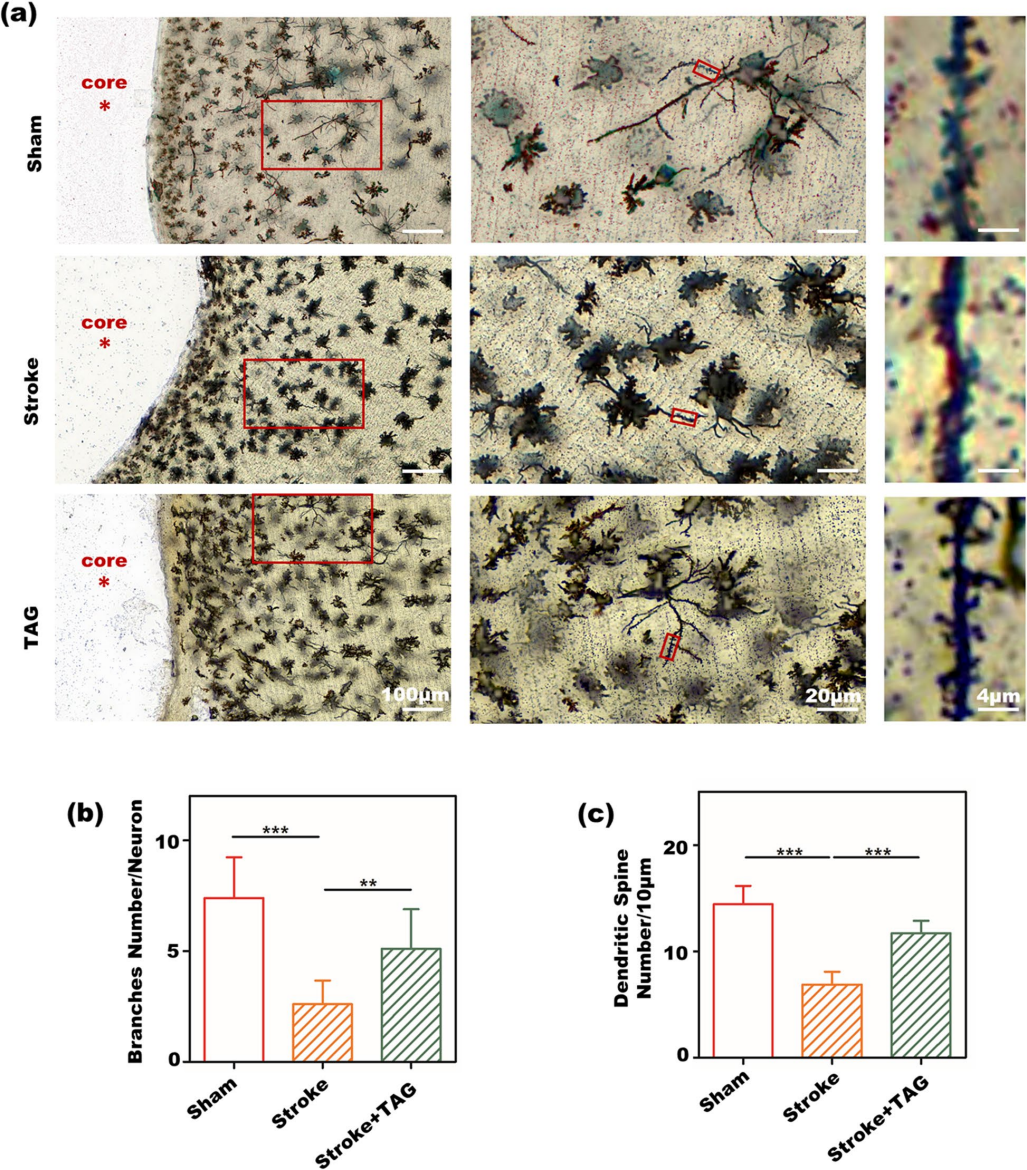
In vivo dendritic plasticity modulated by TA gel **a** images of Golgi staining in the peri-infarct zone of stroke mice; the quantitative results of **b** average branch number in each neuron and **c** average dendritic number within a range of 10 μm around each neuron in IF images of Golgi staining

After ischemic stroke, the cortex adjacent to the infarct cavity undergoes morphological and functional remodeling, including dendritic spine density and dendritic branching, which is important for the formation of new neural connections. To investigate the effects of TA gel treatment on dendritic spine structure after ischemic stroke, we performed Golgi silver impregnation to detect neuronal dendritic spines in the cortical peri-infarct area in sham, stroke, and TAG-treated mice, as shown in Fig. 7. We found that PT stroke induced a significant reduction in the number of dendritic spines and branch numbers, as shown in Fig. 7b and c. Interestingly, TA gel treatment markedly increased dendritic plasticity compared with that in the stroke group, indicating that TA gel treatment can effectively enhance dendritic plasticity in vivo.

**Fig. 8:**
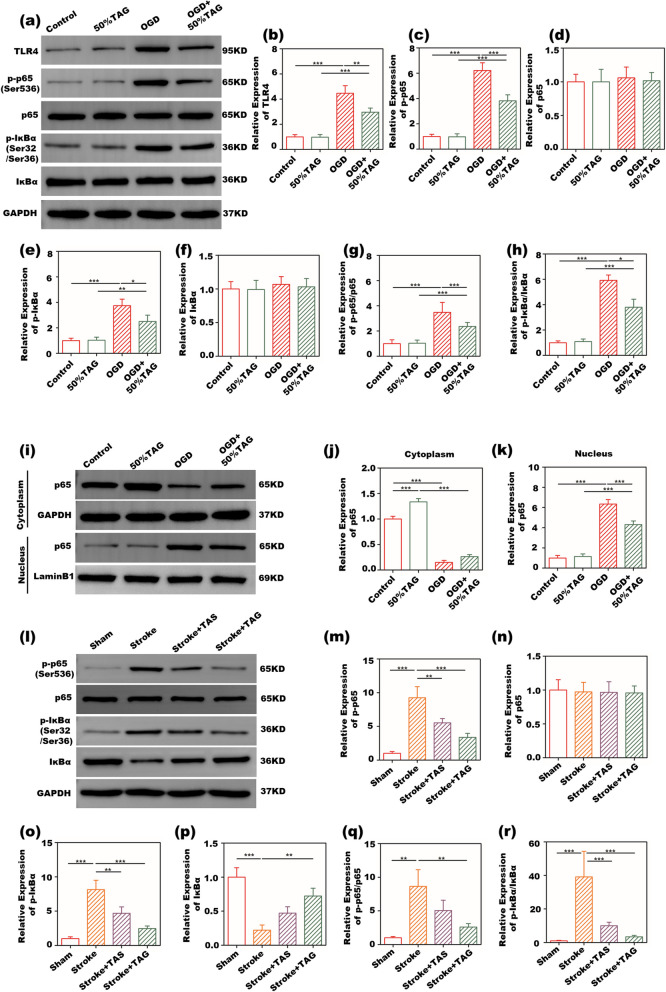
Anti-inflammatory polarization of BV2 cells modulated by the TA gel via the TLR4/NF-κB signaling pathway. **a** Representative WB images of TLR4, p65, phosphorylated p65 (p-p65), IKBα and phosphorylated IKBα (p-IKBα) in vitro; quantitative results of **b** TLR4, **c** p-p65, **d** p65, **e** p-IKBα, f IKBα, **g** p-p65/p65, and **h** p-IKBα/IKBα in vitro; **i** representative WB images of p65 in the cytoplasm and nucleus, quantitative results of p65 in **j** the cytoplasm and **k** the nucleus; **l** representative WB images of p65, p-p65, IKBα and p-IKBα in vivo; quantitative results of **m** p-p65, **n** p65, **o** p-IKBα, **p** IKBα, **q** p-p65/p65, and **r** p-IKBα/IKBα in vivo

TLR4/NF-κB signaling plays an essential regulatory role in stroke-induced proinflammatory microglial polarization [15, 34, 35], we tested whether the TA gel regulated anti-inflammatory microglial polarization by inhibiting the activation of the TLR4/NF-κB signaling pathway in PT stroke models in vitro and in vivo. The protein expression of TLR4 (Fig. 8a and b), phosphorylated IKBα (p-IKBα) (Fig. 8a and e), and phosphorylated p65 (p-p65) (Fig. 8a and c) was markedly increased in the OGD- stimulated group compared to that in the control group. However, the expression of these proteins was significantly decreased in OGD-challenged BV2 cells after treatment with TA gels. Furthermore, the TA gel inhibited OGD-induced increases in the ratios of p-IKBα/IKBα and p-p65/p65. No significant changes in the protein experssion of p65 and IKBα were observed in these groups.

To investigate the nuclear translocation of NF-κB p65 in BV2 microglia after OGD stimulation, we measured the protein levels of p65 in the nucleus and cytoplasm of OGD BV2 cells, (Fig. 8i-k). TA gel treatment dramatically inhibited OGD-induced elevation of p65 in the nucleus, but enhanced p65 expression in the cytoplasm. These data suggest that the TA gel abolished OGD-induced nuclear translocation of p65 from the cytoplasm to the nucleus. Overall, the in vitro results indicate that the inhibitory effect of the TA gel on proinflammatory microglial polarization is associated with suppressing the activation of the TLR4/NF-κB signaling pathway after stroke.

This hypothesis was further supported by in vivo studies examining the expression of NF-κB-related proteins in the brain of PT mice. Consistent with the in vitro results, TA gel treatment dramatically inhibited the phosphorylation of p65 and IKBα and reduced the ratios of p-p65/p65 and p-IKBα/IKBα in the peri-infarct cortex (Fig. 8l-r) at 21 days after PT stroke. These findings indicate that the TA gel dramatically inhibited NF-κB activation in BV2 microglia after PT stroke.

## Discussion

Historically, tannin-containing plants have been used by both Western and Eastern cultures as traditional medicine for various disorders, in particular as an antiphlogistic for inflammatory conditions [36, 37]. Recently, it has been reported that TA can significantly reduce behavioral impairment, oxidative damage, and inflammatory responses caused by traumatic brain injury [38]. Ashabi, et. al. also found that long-term treatment with TA improved hypoperfusion-induced motor deficits and memory dysfunction in a rat model of unilateral common carotid artery occlusion (UCCAO) [39]. Spontaneously, we proposed in Fig. 1 that TA is able to regulate the polarization of microglia by exerting its anti-inflammatory properties.

As mentioned above, an injectable TA gel is the best carrier for TA administration, as it can sustainably release TA in the infarct cavity to regulate microglia polarization on site. The successful formation of the hydrogel is demonstrated by the inverted vital result in Fig. 2a. According to our previous work [40], the rheological property of the TA gel can be modulated by the feed concentration of TA and CMCS. We adopted a suitable feed concentration in this study to assure the injectability of the TA gel according to our experiences. Figure 2b demonstrates that the TA gel can be smoothly injected from a syringe so that it can meet the requirement of intracranial delivery in this study. Furthermore, this feed concentration can give a matchable modulus with the native brain. We measured the moduli of the mouse’s brain in Fig. S8. It showed the modulus of TA gel is matchable with that of native brain tissue. The matchable modulus can benefit the TA gel to play the therapeutic role in enhancing the neuroplasticity. The feed concentration of TA also influenced the degradation rate of the gel. The degradation rate was influenced by many factors such as pH, temperature, gel composition, crosslinking density and so on. The in vitro degradation is carried out at 37°C and pH 7.4 in order to mimic the body environment. As the TA gel is formed by reversible hydrogen bonds, the degradation rate mainly depends on the destruction rate of hydrogen bonds in the hydrogel networks. TA is responsible for the formation of hydrogen bonds in the TA gel, thus higher TA feed concentration usually leads to denser crosslinking network and slower degradation, and vice versa. The cerebrospinal fluid circulation can break the hydrogel bond and lead to the degradation of the gel in vivo, whereas the degradation medium acts as the cerebrospinal fluid to result in the degradation and the release of TA in vitro. The degradation plot in Fig. 2e shows that the TA gel lost 60% of its weight within 16 days. According to the animal test protocol in Fig. 4a, the TA gel injection was arranged on the 7th day after PT-induced stroke, and sacrifice occurred on the 21st day; thus, the TA gel is expected to be able to stay in vivo for approximately two weeks. The in vitro degradation behavior in Fig. 2e indicated that the TA gel can meet this requirement. As discussed above, TA was spontaneously released with the degradation of the TA gel. The in vitro release behavior is shown in Fig. 2f. Similarly, Fig. 2f also shows that the TA gel can assure a sustainable release of TA within approximately two weeks to meet the requirements of the animal test protocol.

Figure 2g and h demonstrate that the hydrogel shows high biocompatibility and low cytotoxicity when cultured with N2a cells. Furthermore, Fig. 3a shows that the TA gel also has high biocompatibility and low cytotoxicity when cultured with BV2 cells by CCK-8 and LDH assays. Since neurons and microglia are two major cells in the brain, these results indicate that the TA gel could be used in the brain without any risks. Many studies have reported the anti-inflammatory ability of TA. Parvez, et al. intraperitoneally injected TA solution into a TBI mouse and found that TA can effectively downregulate the expression of IL-β (interleukin-1 beta) and TNF-ɑ [38]. As IL-β is a typical proinflammatory cytokine secreted by microglia, the results in the literature imply that TA is able to stimulate microglia to decrease the secretion of proinflammatory cytokines and to polarize microglia toward an anti-inflammatory phenotype. Furthermore, Ashabi, et. al. reported that oral administration of TA solution can also downregulate the expression of NF-κB and TNF-ɑ in UCCAO rats [39]. Since the NF-κB pathway is a classic inflammation-related pathway, the suppression and inhibition of the NF-κB pathway can significantly suppress the local inflammatory response. The IF staining and WB results in Fig. 3d-k clearly showed that TAG can significantly decrease CD16 expression and increase CD206 expression in OGD BV2 cells in vitro, exhibiting the potential to alter the activated microglial phenotype in stroke mice. Furthermore, the WB results in Fig. 3h, i and k showed that OGD microglia treated with TAG secreted less proinflammatory IL-β and more anti-inflammatory TGF-β than those without TAG.

The phenotype of microglia plays a vital role in neuroplasticity. Proinflammatory microglia produce destructive cytokines, exacerbating brain damage. In contrast, anti-inflammatory microglia release trophic factors, promoting tissue repair [41]. Figure 3l-p clearly shows that the viability as well as the expression of synaptic plasticity-related proteins such as synaptophysin and PSD95 in OGD N2a cells were significantly upgraded by anti-inflammatory microglia that are regulated by TAG. Thus, the TA gel is able to enhance neuroplasticity in vitro by regulating microglia to polarize them toward an anti-inflammatory phenotype.

Figure 4b shows the in vivo images of the TA gel at different implantation time points. Fluorescence imaging of the mouse brain suggested the presence of TA gel in the mouse brain. The presence of the hydrogel in vivo during the treatment course is a prerequisite for maintaining the local concentration of TA. As the fluorescence intensity decreased with implantation time, the implanted TA gel gradually degraded in vivo. As TA acts as both a building block and a bioactive drug, the degradation of TA gel would release TA simultaneously. The gradual decrease in the fluorescence signal also indicated the sustainable release of TA from the TA gel, which is expected to be able to regulate microglial polarization in vivo. Five groups, including the sham, stroke, CMCS, TAS and TAG groups, were evaluated by WB to investigate their influences on in vivo microglial polarization, as known in Fig. 4c and d. The CMCS group had the same performance as the stroke group and did not show any ability to regulate microglial polarization. Both TAG and TAS exhibited the ability to modulate microglia to express an anti-inflammatory phenotype after stroke, indicating that TA is the active ingredient in the TA gel that regulates microglial polarization. Furthermore, WB also clearly showed that the TA gel has a better ability to regulate microglial polarization than the TA solution. In our previous work, [42] we prepared a multifunctional hydrogel loaded with BDNF (brain-derived neurotrophic factor) and VEGF (vascular endothelial growth factor) for poststroke rehabilitation. It was also found that the multifunctional hydrogel always had better performance than the solution of BDNF and VEGF, in regard to regulating microglial polarization as well as enhancing angiogenesis and neuroplasticity, because the sustainable release behavior of the multifunctional hydrogel can maintain a high growth factor concentration in the peri-infarct area. Similarly, TA gel also has a much better ability to regulate microglial polarization than that of TA solution, in this study, for this reason. The ethological measurements in Fig. 4e and f showed the same tendency as the WB results in Fig. 4c and d. As microglial polarization toward an anti-inflammatory phenotype can enhance neuroplasticity, the consistent results between ethology and microglial polarization are quite reasonable. Moreover, both the balance beam and rotarod tests showed that the TA gel exhibited better ethological results than those of the TA solution, with a significant difference after the 11th day, indicating better functional recovery of TA gel-treated stroke mice. Certainly, better functional recovery also results from the sustainable release of TA in the peri-infarct area. Additionally, since both ethology and WB measurements showed that CMCS had no influence on microglial polarization and subsequent functional recovery, the CMCS solution group was not further investigated in the following in vivo measurements.

Microglial polarization was further investigated by IF staining, as shown in Fig. 5. As resident immune cells in the central nervous system, the primary function of microglia is to maintain normal immune homeostasis. According to the pathology of stroke, the microglia are activated soon after stroke and dynamically polarize from a beneficial anti-inflammatory phenotype toward a neurotoxic proinflammatory phenotype accompanied by morphological and functional changes. At the subacute and chronic stages, microglia mainly possess a proinflammatory phenotype and release proinflammatory cytokines, which exert a detrimental influence on neuroplasticity, such as synaptic remodeling, axonal sprouting, and dendritic spine regeneration. Therefore, strategies aimed at driving anti-inflammatory microglial polarization have emerged as a potential rehabilitative approach to restore neurofunction after stroke. The activation of microglia was clearly demonstrated in Fig. 5 as stroke, and the TAS and TAG groups all possessed higher IBA-1 expression than the sham group. However, the TAG group exhibited lower IBA-1 expression than the stroke and TA solution groups, indicating that TA gel treatment can suppress microglial activation after stroke. Furthermore, TAG showed the lowest percentage of iNOS^+^IBA-1^+^ microglia among total IBA-1^+^ microglia and the highest percentage of CD206^+^IBA-1^+^ microglia among total IBA-1^+^ microglia among all the groups, indicating that there were the fewest proinflammatory microglia and the most anti-inflammatory microglia in the peri-infract zone of mice treated with the TA gel.

As mentioned above, these anti-inflammatory microglia can greatly enhance neuroplasticity to promote poststroke rehabilitation. Furthermore, the TAG group was shown to have lower iNOS/IBA-1 and higher CD206/IBA-1 colocalization than the TAS group, which again demonstrated the advantage of the sustainable release behavior of TA in the TA gel. In addition, microglia were proven to be involved in synaptic plasticity through the release of pro-/anti-inflammatory factors or synaptic pruning after stroke. Anti-inflammatory microglia are known to secrete neuroprotective factors, including BDNF. Thus, shifting pro-inflammatory microglia to an anti-inflammatory phenotype can enhance synaptic plasticity and promote functional outcomes following stroke. Therefore, it is believed that the TA gel can indirectly enhance synaptic plasticity by modulating microglial polarization toward an anti-inflammatory phenotype.

Synaptic plasticity plays a vital role in poststroke rehabilitation, as it is directly related to functional recovery after stroke [43]. PSD95 and Vglut1 double staining was used to investigate synaptic plasticity in the peri-infarct area in this study. PSD95 is the most important and abundant scaffold protein in the postsynaptic membrane, [27, 44] whereas Vglut1 is commonly used as a presynaptic marker [45, 46]. Thus, the connection of these two proteins represents the enhancement of synaptic plasticity [47–49]. As shown in Fig. 6a-d, the amounts of both PSD95 and Vglut1 sharply decreased in stroke mice, which indicated the loss and apoptosis of the cranial nerve due to ischemic onset. Previous studies have demonstrated that anti-inflammatory microglia can improve synaptic plasticity and motor function in a stroke model. Here, the positive puncta of these two proteins in the peri-infract area gradually recovered in both the TAG and TAS groups. As discussed above, TA treatment can effectively induce microglia to express an anti-inflammatory phenotype, and this result supported our previous conclusion that anti-inflammatory microglia can improve synaptic plasticity. Furthermore, mice treated with TAG exhibited more positive particles of the two proteins than those treated with TAS. Thus, the TA gel possessed a better ability to enhance poststroke synaptic plasticity. Moreover, as the colocalization of PSD95 and Vglut1 reflects the synaptic connection, it can better characterize synaptic plasticity in the peri-infarct area. Figure 6 shows that the colocalization of PSD95 and Vglut1 was markedly decreased after stroke and recovered to some extent after treatment with both TAG and TAS. The TA gel treatment still induced more synaptic connections than the TA solution.

It was reported that synaptic connections are directly related to motor functions [27]. Thus, the best synaptic connection of the TAG in Fig. 6 supported the ethological results in Fig. 4 that TAG has the best functional recovery after stroke. Furthermore, it also showed that sustainable release of TA in vivo by the TAG can achieve better synaptic connection than one-time intracranial administration by the TAS group. The WB results in Fig. 6e-g further demonstrated that TAG has the best ability to enhance synaptic plasticity since it possessed the highest synaptophysin and PSD95 expression in vivo. Hence, based on the above results of PSD95 and Vglut1 containing as well as the WB results of synaptophysin and PSD95 expression, it can be concluded that the TA gel can promote synaptic plasticity by sustainably releasing TA to regulate microglial polarization toward an anti-inflammatory phenotype in vivo.

Dendritic spine densities, an independent line of evidence for poststroke recovery, are one of the neural structural and functional plasticity factors recognized as the cornerstone of functional restoration after stroke [50]. Dendritic plasticity plays a crucial role in neural network rebuilding in the peri-infarct area. The increase in dendritic spine densities occurs in the surviving neurons next to the infarct cavity and contributes to rebuilding neural networks taking over the function of the lost neurons [51]. Here, we detected significant changes in dendritic spine densities in the peri-infarct area in the cortex based on Golgi staining, as shown in Fig. 8. After PT stroke, dendritic spine density in the peri-infarct region was greatly reduced, whereas the TA gel treatment remarkably reversed the stroke-induced decrease in dendritic spine density.

Thus, TA gel-afforded functional restoration might be partially attributed to dendritic spine rebuilding. In line with our results, several studies have demonstrated that poststroke functional outcomes were associated with increases in dendritic spine density [52, 53]. Consequently, it can be concluded that TA gel treatment after stroke can significantly enhance neuroplasticity in the peri-infarct zone, as PSD95 and Vglut1 containing as well as the WB results of synaptophysin and PSD95 proved the enhancement of synaptic plasticity, and Golgi staining demonstrated the improvement of dendritic plasticity.

Several transcription factors, such as STAT1, [15] NF-κB p65, [14] and IRF5, [54] were demonstrated to be involved in proinflammatory microglial polarization after stroke. Therapeutic approaches inhibiting the activation of these transcription factors to suppress proinflammatory microglial polarization might provide a novel restorative strategy for stroke. Zhao et al. designed a nanoparticle (NP) in which ferulic acid diacid with an adipic acid linker (FAA) and tannic acid (TA) were used as shell and core molecules, respectively [55]. They found that NP treatment significantly inhibited NF-κB activation and reduced IBA-1 expression in α-synuclein-stimulated microglia. Furthermore, the anti-inflammatory effects of TA were associated with the suppression of NF-κB pathway activation in LPS-induced BV2 microglia [56].

In this study, we have indicated that TA gel shifted microglial polarization toward an anti-inflammatory phenotype following PT stroke. The mechanisms underlying TA gel-mediated microglial polarization after stroke remain to be elucidated. Our western blot data showed that the TA gel decreased the protein expression of TLR4 and NF-κB related proteins, including p-IKBα and p-p65, in OGD-induced microglia. Additionally, TA gel administration reduced the translocation of NF-κB p65 from the cytoplasm to the nucleus, thereby alleviating proinflammatory microglial polarization. Our findings demonstrated that TA gel switched the proinflammatory microglial phenotype to an anti-inflammatory state, at least partially, by blocking NF-κB activation.

## Conclusions

TA can act as both a building block to form an injectable TA gel with CMCS and a bioactive drug to promote poststroke rehabilitation in this study. These results showed that the TA gel can polarize OGD BV2 cells toward an anti-inflammatory phenotype in vitro. Then, the expression of synaptophysin and PSD95 in OGD N2a cells was effectively recovered by TA gel-treated OGD BV2 cells, implying the ability of the TA gel to enhance in vitro neuroplasticity by modulating microglial polarization. After being injected into the stroke cavity of mice, the ability of the TA gel to modulate microglial polarization and subsequently enhance neuroplasticity was further proven by IF staining, WB and Golgi staining. The recovery of the motor function of stroke mice was illustrated by the ethological measurements, including rotarod and balance beam. Additionally, it was proven both in vitro and in vivo that the TA gel modulates microglia to polarize them toward an anti-inflammatory phenotype via the NF-κB pathway. Consequently, the injectable TA gel can promote poststroke rehabilitation in stroke mice by modulating microglial polarization to enhance neuroplasticity.

### Supplementary Information

**Additional file 1:** **Fig. S1.** Chemical reaction equation of TA and CMCS. **Fig. S2.** Immunofluorescence images of N2a cells by live/dead assay. **Fig. S3.** Immunofluorescence images of CD16 and CD206 staining in BV2 cells after OGD. **Fig. S4.** Chemical reaction equation of Cy5-NHS and CMCS. **Fig. S5.** Immunofluorescence spectrum of CMCS-Cy5. **Fig. S6.** SEM image of immunofluorescent TA gel formed by TA and CMCS-Cy5. **Fig. S7.** Standard curve of TA in aqueous solution. **Fig. S8.** The rheological curves of the mouse’s brain.

## Data Availability

All data generated or analysed during this study are included in this published article and its supplementary information files.
