# Supplementary material for: Nanoarchitectonics of tannic acid based injectable hydrogel regulate the microglial phenotype to enhance neuroplasticity for poststroke rehabilitation
Source: Biomater Res. 2023 Oct 31;27:108. doi: 10.1186/s40824-023-00444-0 (PMC10617113; doi:10.1186/s40824-023-00444-0)
Supplement: Supplementary file 1 — Additional file 1: Fig. S1. Chemical reaction equation of TA and CMCS. Fig. S2. Immunofluorescence images of N2a cells by live/dead assay. Fig. S3. Immunofluorescence images of CD16 and CD206 staining in BV2 cells after OGD. Fig. S4. Chemical reaction equation of Cy5-NHS and CMCS. Fig. S5. Immunofluorescence spectrum of CMCS-Cy5. Fig. S6. SEM image of immunofluorescent TA gel formed by TA and CMCS-Cy5. Fig. S7. Standard curve of TA in aqueous solution. Fig. S8. The rheological curves of the mouse’s brain. [file 40824_2023_444_MOESM1_ESM.docx]

Supporting Information

**Nanoarchitectonics of tannic acid based injectable hydrogel regulate the microglial phenotype to enhance neuroplasticity for poststroke rehabilitation**

Zongjian Liu^1#^, Shulei Zhang^4#^, Yuanyuan Ran^1#^, Huimin Geng^3*^, Fuhai Gao^1^, Guiqin Tian^1^, Zengguo Feng^4^, Jianing Xi^1^, Lin Ye^4*^and Wei Su^2*^

1. Beijing Rehabilitation Hospital, Capital Medical University, Beijing 100044, China

2. Beijing Tsinghua Chang Gung Hospital, School of Clinical Medicine, Tsinghua University, Beijing 102218, China

3. Department of Neurosurgery, Qilu Hospital of Shandong University, Shandong University, Jinan, Shandong 250012, China.

4. School of Materials Science and Engineering, Beijing Institute of Technology, Beijing 100081, China

# Contributed equally

*Corresponding authors：

E-mail: [yelin@bit.edu.cn](mailto:yelin@bit.edu.cn) (L.Y.), [swa01179@btch.edu.cn](mailto:swa01179@btch.edu.cn) (W. S.), [hmgeng@sdu.edu.cn (H](mailto:hmgeng@sdu.edu.cn%20(H). G.)


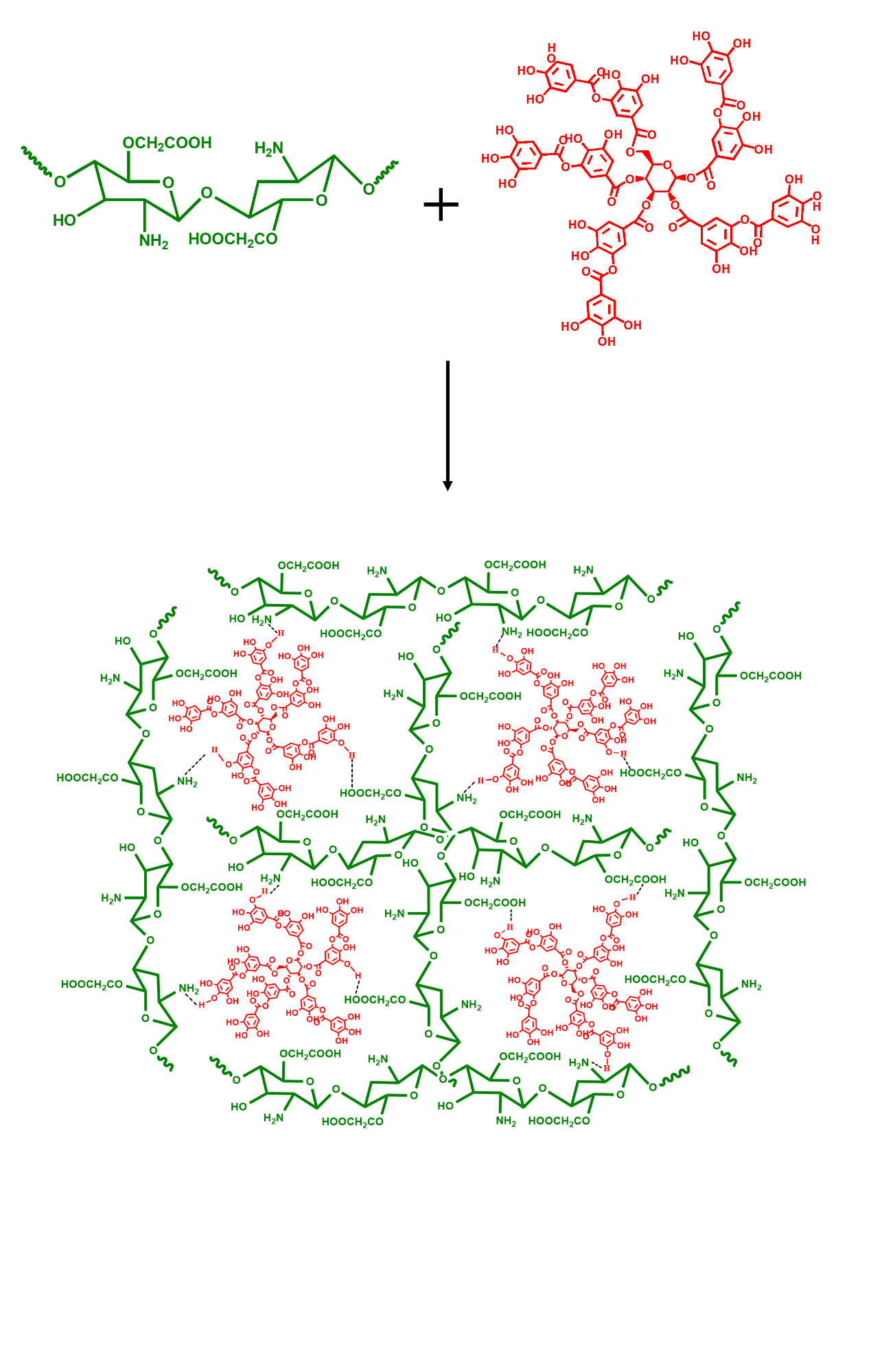


**Fig.S1 Chemical reaction equation of TA and CMCS.**


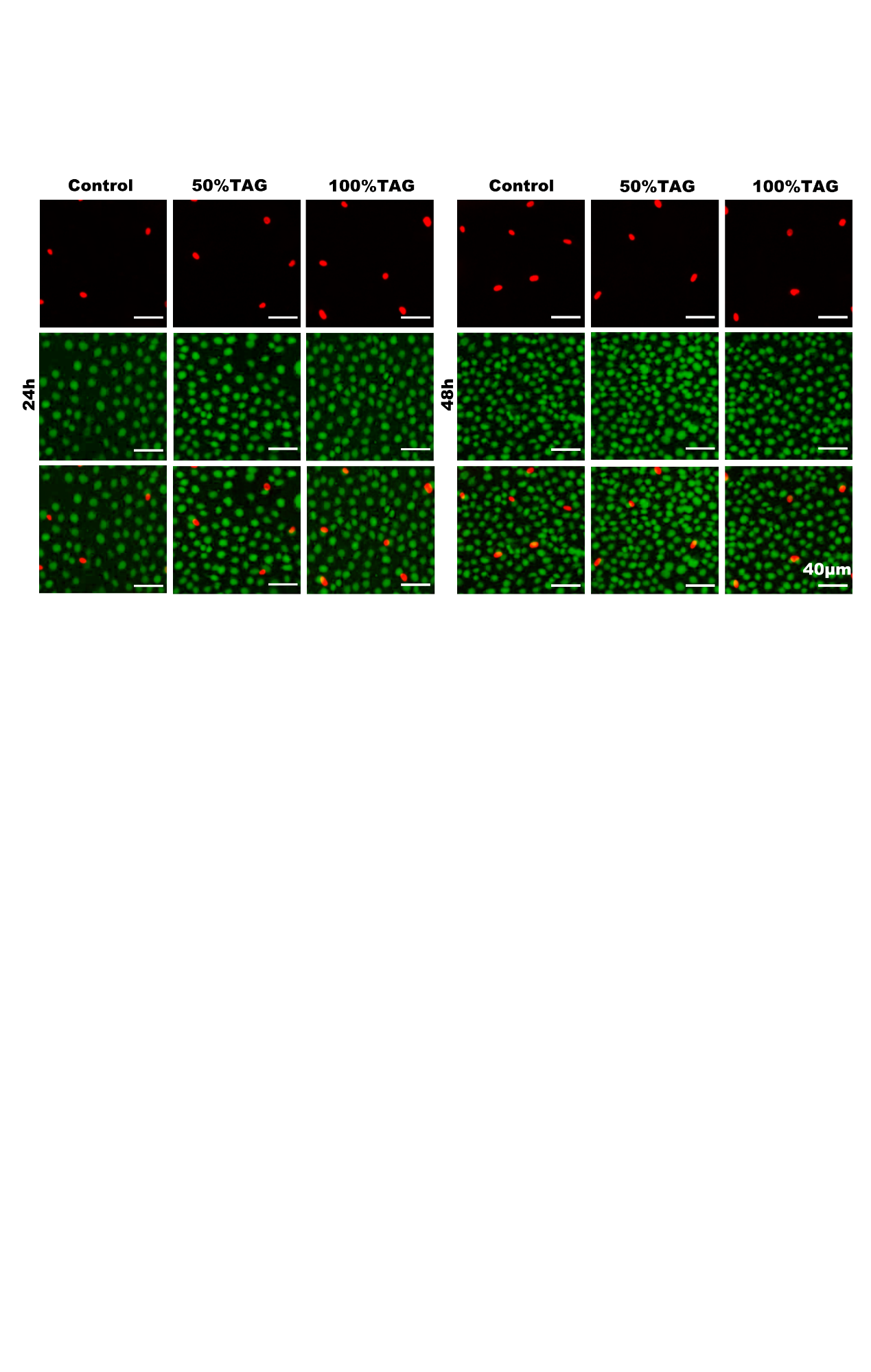


**Fig.S2 Immunofluorescence images of N2a cells by live/dead assay**


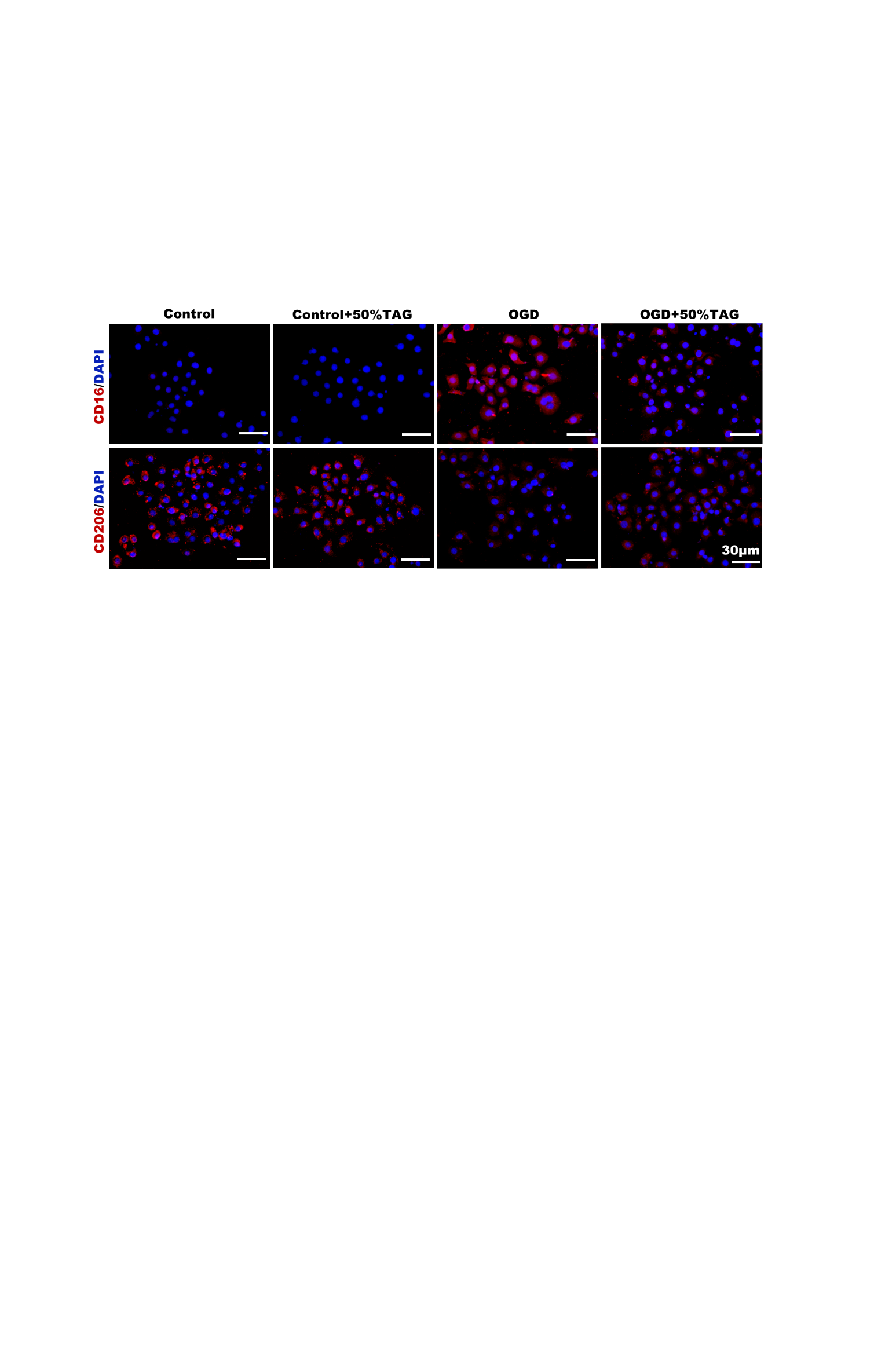


**Fig.S3 Immunofluorescence images of CD16 and CD206 staining in BV2 cells after OGD**


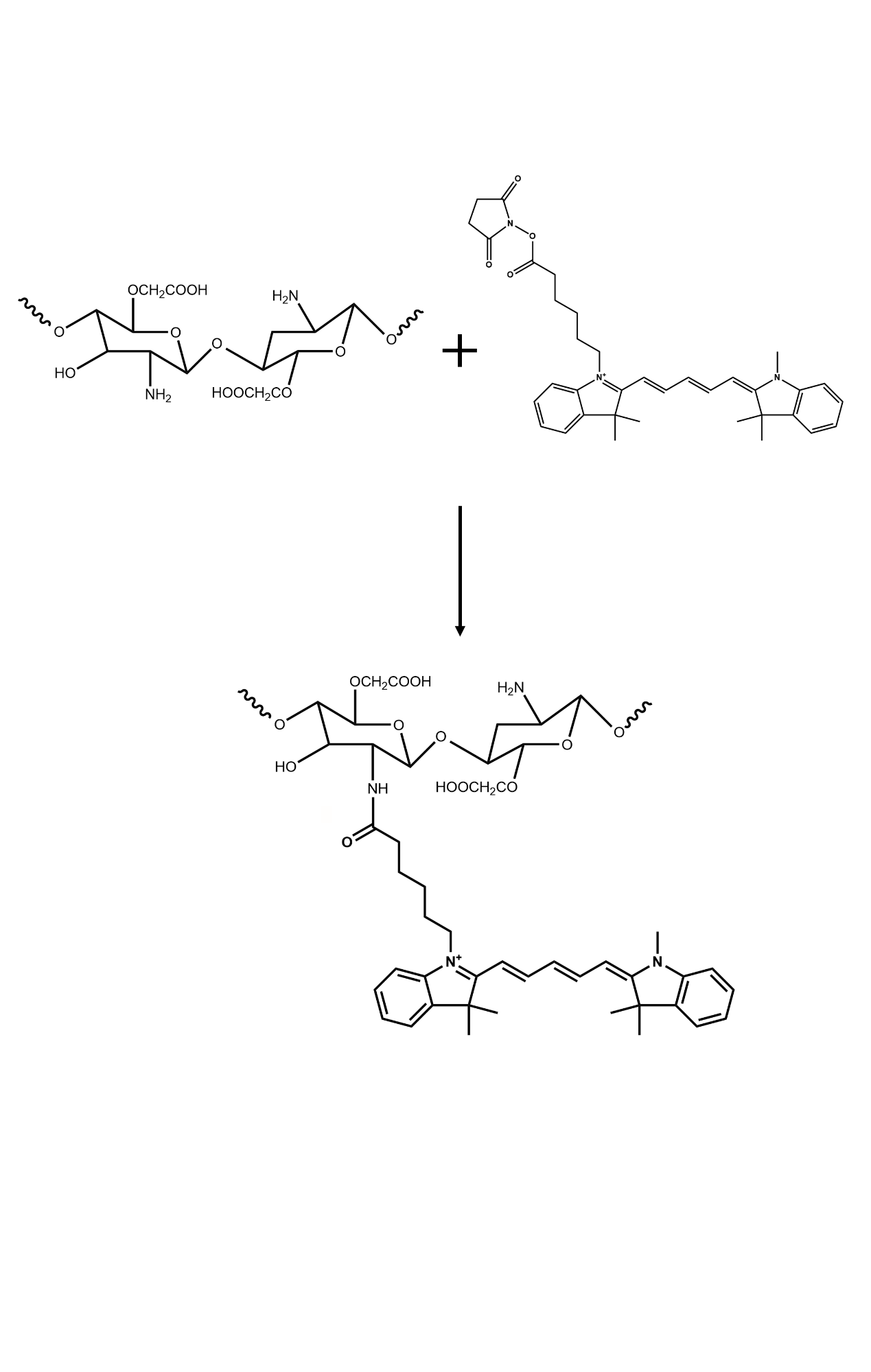


**Fig.S4** **Chemical reaction equation of Cy5-NHS and CMCS**


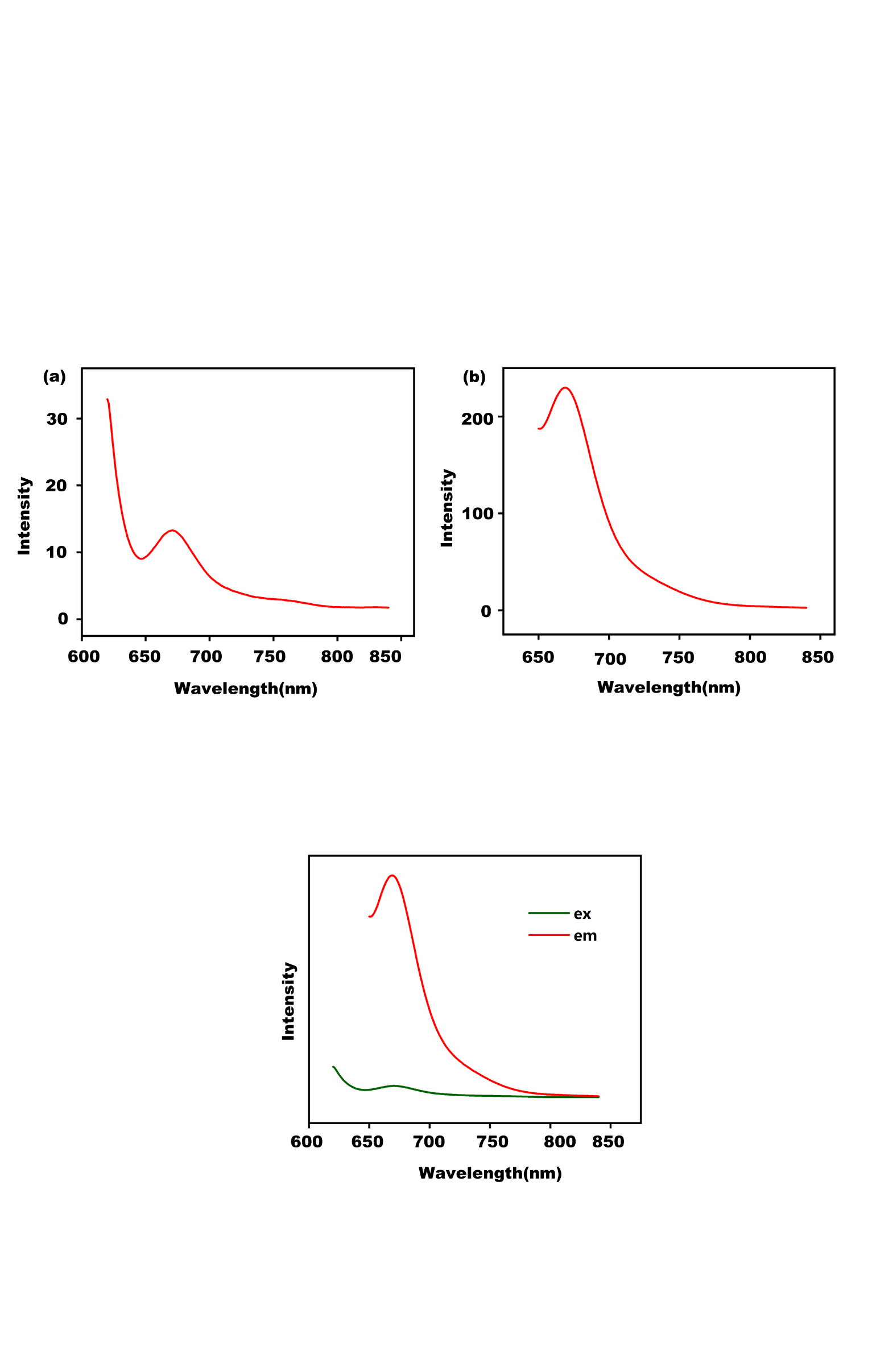


**Fig.S5** **Immunofluorescence spectrum of CMCS-Cy5**


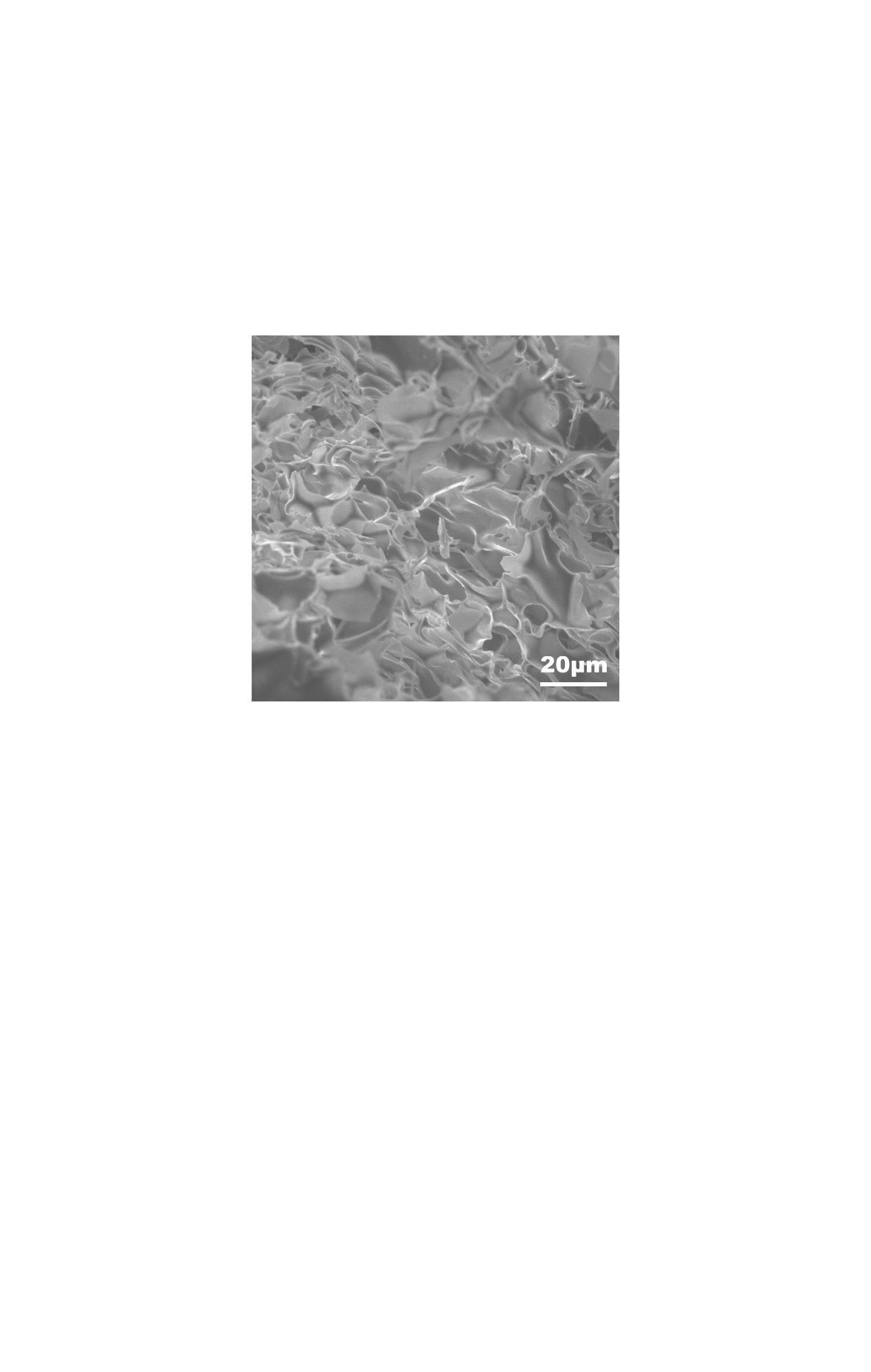


**Fig.S6** **SEM image of Immunofluorescent TA gel formed by TA and CMCS-Cy5**


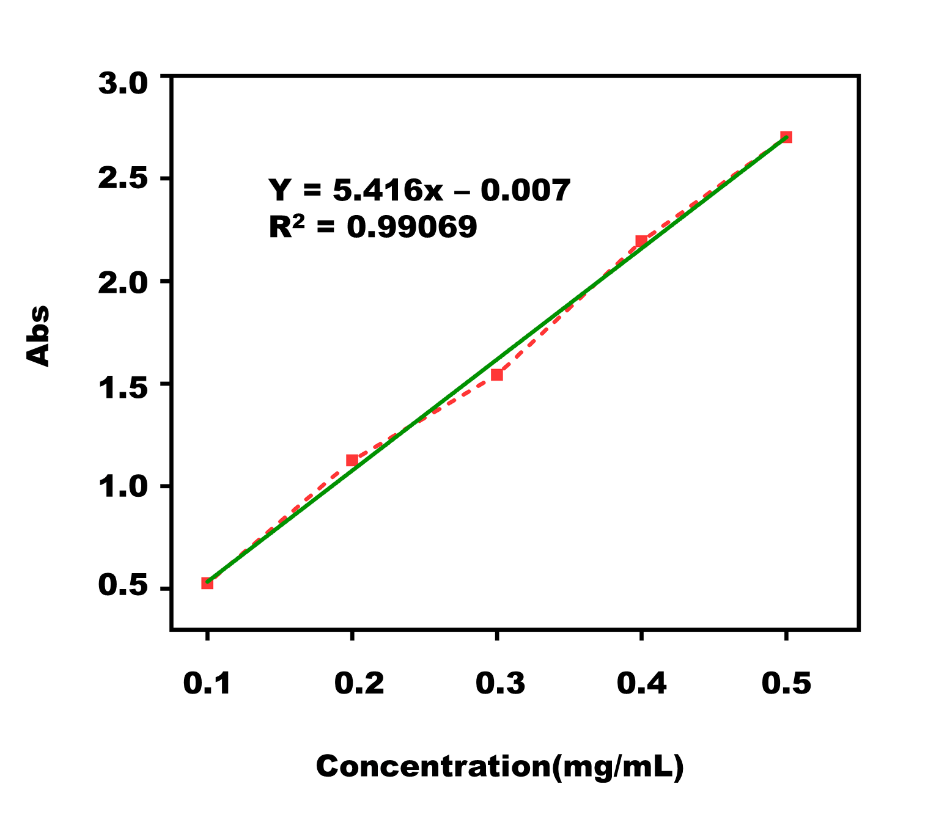


**Fig.S7** **Standard curve of TA in aqueous solution**


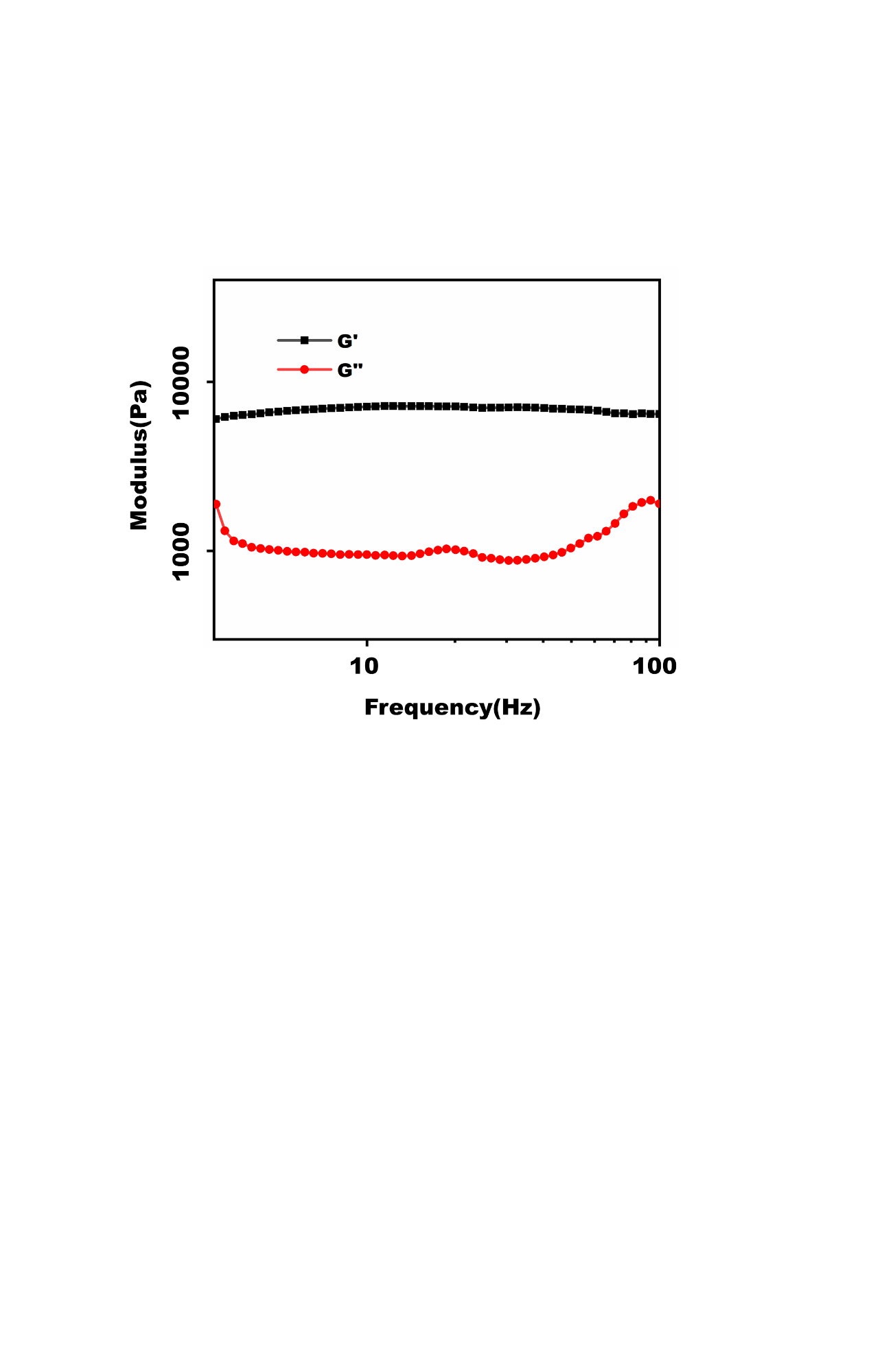


**Fig. S8 The rheological curves of the mouse’s brain.**
